# Multidimensional definition of the interferonopathy of Down syndrome and its response to JAK inhibition

**DOI:** 10.1126/sciadv.adg6218

**Published:** 2023-06-28

**Authors:** Matthew D. Galbraith, Angela L. Rachubinski, Keith P. Smith, Paula Araya, Katherine A. Waugh, Belinda Enriquez-Estrada, Kayleigh Worek, Ross E. Granrath, Kohl T. Kinning, Neetha Paul Eduthan, Michael P. Ludwig, Elena W. Y. Hsieh, Kelly D. Sullivan, Joaquin M. Espinosa

**Affiliations:** ^1^ Linda Crnic Institute for Down Syndrome, University of Colorado Anschutz Medical Campus, Aurora, CO, USA.; ^2^ Department of Pharmacology, University of Colorado Anschutz Medical Campus, Aurora, CO, USA.; ^3^ Department of Pediatrics, Section of Developmental Pediatrics, University of Colorado Anschutz Medical Campus, Aurora, CO, USA.; ^4^ Department of Immunology and Microbiology, University of Colorado Anschutz Medical Campus, Aurora, CO, USA.; ^5^ Department of Pediatrics, Division of Allergy/Immunology, University of Colorado Anschutz Medical Campus, Aurora, CO, USA.; ^6^ Department of Pediatrics, Section of Developmental Biology, University of Colorado Anschutz Medical Campus, Aurora, CO, USA.

## Abstract

Individuals with Down syndrome (DS) display chronic hyperactivation of interferon signaling. However, the clinical impacts of interferon hyperactivity in DS are ill-defined. Here, we describe a multiomics investigation of interferon signaling in hundreds of individuals with DS. Using interferon scores derived from the whole blood transcriptome, we defined the proteomic, immune, metabolic, and clinical features associated with interferon hyperactivity in DS. Interferon hyperactivity associates with a distinct proinflammatory phenotype and dysregulation of major growth signaling and morphogenic pathways. Individuals with the highest interferon activity display the strongest remodeling of the peripheral immune system, including increased cytotoxic T cells, B cell depletion, and monocyte activation. Interferon hyperactivity accompanies key metabolic changes, most prominently dysregulated tryptophan catabolism. High interferon signaling stratifies a subpopulation with elevated rates of congenital heart disease and autoimmunity. Last, a longitudinal case study demonstrated that JAK inhibition normalizes interferon signatures with therapeutic benefit in DS. Together, these results justify the testing of immune-modulatory therapies in DS.

## Teaser

Interferon hyperactivity shapes the pathophysiology of Down syndrome and is attenuated by JAK inhibition.

## INTRODUCTION

Down syndrome (DS) is caused by triplication of chromosome 21 (chr21), i.e., trisomy 21 (T21), the most common chromosomal abnormality in the human population (*1*). Individuals with DS display various developmental phenotypes, including delayed growth and a distinct neurocognitive profile (*2*). The notable increase in the life expectancy of individuals with DS in the past five decades has led to the realization that T21 causes a unique spectrum of co-occurring conditions across the life span, with decreased prevalence of some medical conditions and increased prevalence of others relative to the general population (*2*). Newborns with DS are at high risk of congenital heart defects (CHD), Hirschsprung’s disease, and transient myeloproliferative disorder, and children with DS are predisposed to develop leukemia, autism, and seizure disorders (*3*). Across the life span, individuals with DS are highly prone to developing autoimmune disorders, most prominently autoimmune thyroid disease (AITD), celiac disease, and autoimmune skin conditions (*4*, *5*). The strong immune dysregulation characteristic of DS also manifests through more severe complications from respiratory infections, including coronavirus disease 2019 (COVID-19) (*5*, *6*). Adults with DS display much lower rates of most solid malignancies as well as hypertension and atherosclerosis (*5*, *7*). Later in life, individuals with DS are strongly predisposed to develop Alzheimer’s disease (*8*). Despite substantial research efforts, with a few notable exceptions, the mechanisms by which T21 causes the myriad developmental and clinical hallmarks of DS await elucidation. Therefore, research in this area would benefit not only people with DS but also the general population affected by the medical conditions that are modulated by T21. For example, studies of the amyloid precursor protein gene (*APP*) encoded on chr21 have enabled a better understanding of the role of *APP* triplication versus other accompanying processes in the etiology of Alzheimer’s disease in both the general population and those with DS (*8*).

Within this framework, previous analyses of the transcriptome, proteome, metabolome, and immune cell repertoire of individuals with DS demonstrated that T21 consistently activates the interferon (IFN) transcriptional response across multiple cell types (*9*, *10*), concurrent with changes in the circulating proteome indicative of chronic autoinflammation (*11*), global immune remodeling associated with hypersensitivity to type I IFN stimulation and elevated Janus kinase/signal transducer and activator of transcription (JAK/STAT) signaling (*10*, *12*, *13*), and activation of the IFN-inducible kynurenine pathway, leading to production of neurotoxic tryptophan catabolites (*14*, *15*). These findings support the hypothesis that DS can be understood, in part, as an atypical interferonopathy associated with triplication of four IFN receptor (IFNR) genes encoded on chr21 (i.e., *IFNAR1*, *IFNAR2*, *IFNGR2*, and *IL10RB*) (*10*). Despite these advances, the contributions of chronic IFN hyperactivity to the myriad developmental and clinical features of DS remain to be elucidated. Experiments in mouse models of DS demonstrated that normalization of the *IFNR* gene copy number can fully or partially rescue several phenotypes in these animals, including impaired fetus growth and neuronal viability (*16*), as well as lethal antiviral responses, congenital heart malformations, craniofacial anomalies, early developmental delays, and cognitive impairments (*17*). Although these results support the notion that an interferonopathy underlies much of the pathophysiology of DS, the multiple impacts of IFN hyperactivity on the development, physiology, metabolism, clinical risk profiles, and accelerated aging of persons with DS await elucidation. Furthermore, the molecular and cellular mechanisms by which dysregulated IFN signaling may cause these effects are also unknown.

To advance the understanding of the interferonopathy of DS, we completed an integrated multiomics analysis of IFN signaling in a large cohort of individuals with T21 compared to euploid controls. Using IFN scores derived from the whole blood transcriptome, we defined associations between levels of IFN hyperactivity and the proteome, immune cell profile, metabolome, and clinical variables. This effort revealed that the interferonopathy of DS is clearly of a mixed type, being strongly associated with circulating protein levels of IFNγ (IFNG, type II IFN) and IFNλ1 (IFNL1, type III IFN). Furthermore, IFN hyperactivity is associated with a distinct immune profile in DS marked by elevation of acute phase proteins and key cytokines strongly tied to the development of autoimmunity, such as interleukin-6 (IL-6) and tumor necrosis factor–α (TNFα). IFN hyperactivity accompanies the global proteomic changes observed in DS, being significantly associated with dysregulation of growth factor signaling and developmental pathways, as well as complement, coagulation, and fibrinolysis cascades. Individuals with DS with the highest IFN activity have the most marked shifts in the peripheral immune system, including a reduction in naïve T cell subsets along with enrichment of cytotoxic T cells, B cell depletion, and monocyte activation. At the metabolic level, IFN hyperactivity is associated with dysregulation of tryptophan catabolism, fatty acid metabolism, and central carbon metabolism. Furthermore, IFN hyperactivity associates with a specific pattern of co-occurring conditions in DS, including increased prevalence of CHD and AITD. Through a longitudinal case study, we demonstrate that JAK inhibition normalizes IFN signaling in a research participant with DS in a reversible fashion and without overt immune suppression. Last, we demonstrate that JAK inhibition attenuates global dysregulation of gene expression and decreases IFN signatures across multiple organ systems in a mouse model of DS. Together, these results advance our understanding of hyperactive IFN signaling in DS while justifying the testing of therapeutic interventions targeting the IFN pathway to improve health outcomes in this population.

## RESULTS

### Hyperactive IFN signaling in DS occurs without overt IFN overproduction

To investigate the multidimensional impacts of IFN hyperactivity among individuals with DS, we completed a multiomics analysis in a research cohort of 502 participants from the Human Trisome Project (HTP) biobank, 356 with T21 versus 146 euploid controls, along with deep annotation of demographics and clinical data (fig. S1A and data S1A; see Materials and Methods). Blood samples were analyzed by whole blood transcriptome analysis via RNA sequencing (RNA-seq), plasma proteomics using the SOMAscan platform, inflammatory marker profiling with multiplexed immunoassays using Meso Scale Discovery (MSD) technology, plasma metabolomics via mass spectrometry, and immune cell phenotyping via mass cytometry (fig. S1, A to F, and data S1, B to F; see Materials and Methods). For a core set of ~400 participants, ~300 of them with T21, the datasets were generated from the exact same blood draw, enabling effective cross-platform analyses (fig. S1G).

**Fig. 1.**
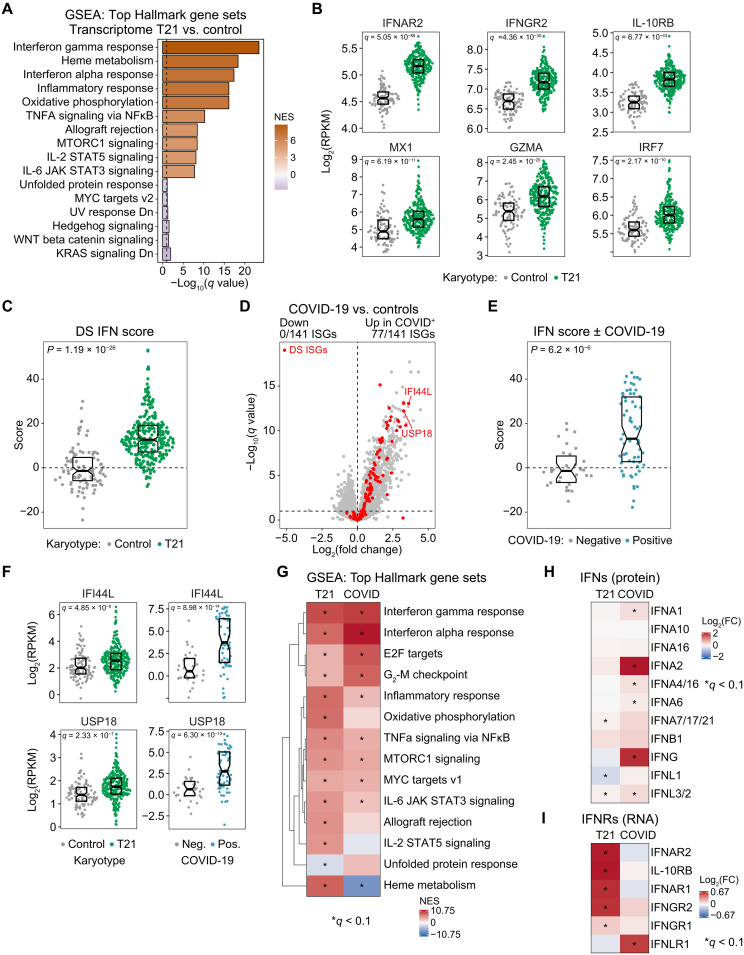
Comparative analysis of the IFN response in DS versus COVID-19. (**A**) Barplot summarizing results of GSEA of gene expression changes in whole blood of individuals with T21 (*n* = 304) versus euploid controls (D21; *n* = 96). Bars are color-coded by positive (orange) or negative (purple) normalized enrichment score (NES) values. (**B**) Sina plots displaying mRNA levels for IFNRs encoded on chr21 (*IFNAR2*, *IFNGR2*, and *IL10RB*), *MX1*, an ISG encoded on chr21, and *GMZA* and *IRF7*, encoded elsewhere in the genome. (**C**) Sina plot displaying DS IFN scores calculated from the expression of the top 18 ISGs induced in the whole blood transcriptome of individuals with T21 (fold change > 1.5, *q* < 0.1). (**D**) Volcano plots summarizing whole blood transcriptome analysis of patients hospitalized with acute COVID-19 (*n* = 73) versus COVID-19–negative controls (*n* = 32). A total of 141 ISGs elevated in whole blood transcriptome of individuals with DS are highlighted in red. (**E**) Sina plot displaying DS IFN scores for acute COVID-19 cases versus COVID-19–negative controls. (**F**) Sina plots displaying the induction of canonical ISGs (*IFI44L* and *USP18*) in DS and COVID-19. (**G**) Heatmap comparing the GSEA NES for whole blood transcriptome changes in DS (T21) versus COVID-19. Color coding indicates transcriptional signatures enriched among up-regulated (red) or down-regulated (blue) genes, as defined by positive or negative NES values. (**H**) Heatmap comparing plasma protein fold changes for specific IFNs in individuals with T21 versus euploid controls (left) and patients with COVID-19 versus COVID-19–negative controls (right). (**I**) Heatmap comparing mRNA fold changes for individual IFNRs measured in the whole blood transcriptome of individuals with T21 versus euploid controls (left) and patients with COVID-19 versus COVID-19–negative controls (right). Boxes in sina plots represent interquartile ranges and medians, with notches approximating 95% confidence intervals. See also figs. S1 and S2. RPKM, reads per kilobase per million mapped reads.

It is well-documented that IFN signaling is hyperactive in DS, which could be explained in part by the presence of type I, II, and III *IFNR* genes on chr21 (*9*, *10*, *12*, *13*, *15*, *17*). In agreement with previous reports using much smaller cohorts (*9*, *12*, *13*, *15*), gene set enrichment analysis (GSEA) of the whole blood transcriptome dataset identified the IFN gamma response, IFN alpha response, and inflammatory response hallmark gene sets among the top positively enriched signatures in DS (Fig. 1A, fig. S2A, and data S2A). Comparison of up-regulated genes comprising the IFN gamma, IFN alpha, and inflammatory response GSEA signatures highlights both common and unique genes (fig. S2B), with 141 known IFN-stimulated genes (ISGs) across both the IFN alpha and gamma signatures identified as significantly up-regulated in DS (fig. S2B). Therefore, the IFN signature associated with DS cannot be simply equated to type I or type II IFN signaling and is likely a mix of both. Expectedly, all four *IFNR* genes encoded on chr21 are significantly overexpressed in T21 (i.e., *IFNAR1,IFNAR2*, *IFNGR2*, and *IL10RB*) (Fig. 1B, fig. S2C, and data S1B). Of the two IFNRs encoded elsewhere in the genome, *IFNGR1* was mildly elevated in T21 samples, whereas *IFNLR1* was not (fig. S2C). Key ISGs up-regulated in DS include some encoded on chr21 (e.g., *MX1* and *MX2*) and many more encoded elsewhere in the genome (e.g., *GZMA*, *IRF7*, *IFITM3*, and *CXCL10*) (Fig. 1B and fig. S2C).

IFN transcriptional scores are commonly used as biomarkers of disease severity and response to treatment in IFN-driven conditions such as systemic lupus erythematosus (SLE), type I interferonopathies, and diverse autoinflammatory conditions and are commonly calculated as the sum of *z* scores for specific sets of elevated ISGs, varying in number from 5 to 50+ genes (*18*, *19*). Thus, we calculated DS IFN scores using ISGs significantly elevated in T21 at least 1.5-fold and that are not encoded on chr21 (18 genes; fig. S2D and data S2B). We specifically excluded ISGs encoded on chr21 from this gene signature, whose elevation is likely because of increased gene dosage, with the aim of focusing instead on the downstream IFN transcriptional response. Expectedly, individuals with DS displayed greatly elevated IFN scores compared to euploid controls, with a wide range of interindividual variability (Fig. 1C). Notably, IFN scores did not vary significantly by sex or age (fig. S2, E and F). Calculating the DS IFN scores with larger numbers of ISGs produced similar results, indicating that the 18 ISG score is adequate to monitor the range of IFN hyperactivity in DS (fig. S2, G and H).

To gain further insight into the nature of IFN hyperactivity in DS, we interrogated datasets that our team generated via the COVIDome Project using a similar experimental pipeline, including identical whole blood transcriptome analysis of 73 hospitalized patients with COVID-19 versus 32 COVID-19–negative controls, none with DS (*20*–*22*). IFN signaling is strongly activated in COVID-19 as part of the antiviral response to severe acute respiratory syndrome coronavirus 2 (SARS-CoV-2), with IFN alpha and gamma response signatures being the top gene sets enriched in the whole blood transcriptome of hospitalized patients with COVID-19 (*22*). Notably, more than half of the 141 ISGs significantly elevated in DS are also significantly elevated in COVID-19 (Fig. 1D). When calculating IFN scores for the COVIDome Project samples using the exact same set of 18 ISGs, we observed that these scores were elevated in COVID-19 to a similar extent as seen in DS, also with strong interindividual variability (Fig. 1E). For example, *IFI44L* and *USP18* are commonly up-regulated in both DS and COVID-19 (Fig. 1F). Comparative GSEA of the top transcriptional signatures in the two conditions revealed a clear similarity in affected pathways, with a few notable differences (Fig. 1G). Although both conditions involve elevation of IFN alpha and IFN gamma responses, IFN alpha is more strongly enriched in COVID-19. Other differences include a transcriptional signature of IL-2 STAT5 signaling elevated only in DS and a signature of heme metabolism being elevated in DS but repressed in COVID-19.

We previously reported significant elevation of plasma protein levels of six different type I, II, and III IFNs in patients with COVID-19 (IFNA1, IFNA2, IFNA4/16, IFNA6, IFNG, and IFNL3/2) and that IFN scores are associated mostly with levels of IFNA2 and IFNG in COVID-19 (*22*). Notably, in DS, we observed only mild elevation of IFNA7/17/21 and IFNL3/2, along with a mild decrease in IFNL1 levels (Fig. 1H). Conversely, none of the *IFNRs* encoded on chr21 are elevated at the RNA level in patients with COVID-19, who instead display up-regulation of *IFNLR1* (Fig. 1I). Therefore, although IFN signaling is hyperactive in both DS and COVID-19, with similar activation of IFN transcriptional signatures, this phenomenon is linked to *IFNR* overexpression in DS versus IFN protein induction in COVID-19.

Together, these results indicate that T21 causes IFN hyperactivity even in the absence of obvious viral infection and without massive elevation of IFNs, leading rather to a mixed-type interferonopathy associated with *IFNR* overexpression. Furthermore, the DS IFN score provides a tool to define associations between hyperactive IFN signaling, other physiological processes dysregulated in DS, co-occurring conditions, and responses to therapeutic interventions.

### IFN hyperactivity uncovers a distinct proinflammatory subtype in DS

**Fig. 2.**
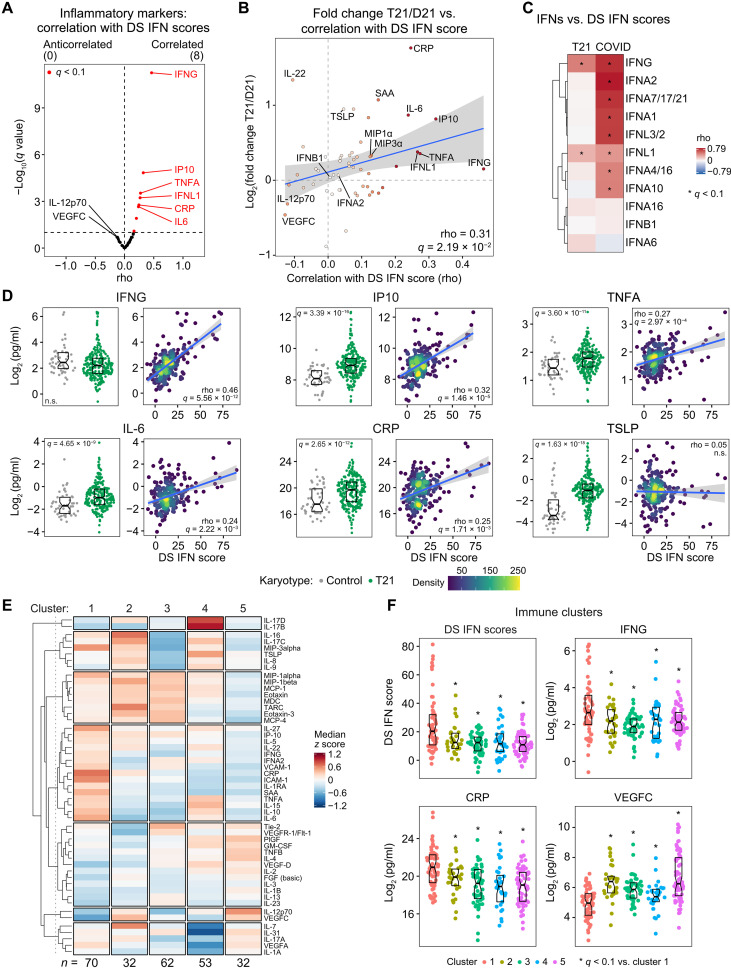
IFN hyperactivity associates with a distinct proinflammatory immune profile in DS. (**A**) Volcano plot displaying Spearman correlation scores (rho) between transcriptional DS IFN scores and 54 inflammatory markers measured by MSD assays in plasma from 249 individuals with T21. Significance is defined as *q* < 0.1 [10% false discovery rate (FDR)]. (**B**) Scatterplot comparing the effects of T21 on the levels of 54 inflammatory markers (*y* axis) with their correlation to DS IFN scores (*x* axis). Color intensity of points increases with absolute rho value; the blue line represents a linear fit, with 95% confidence interval in gray. (**C**) Heatmap displaying Spearman correlation rho values for DS IFN scores versus plasma protein levels of 12 IFNs measured by MSD or SOMAscan assays in individuals with DS (*n* = 249) or euploid patients hospitalized with acute COVID-19 (*n* = 71). Asterisks indicate *q* < 0.1. (**D**) Sina plots (left) displaying the effects of T21 on levels of inflammatory markers and scatterplots (right) displaying relationships to DS IFN scores among those with T21 (with Spearman correlation rho and *q* values). Points are colored by density; blue lines represent linear fit, with 95% confidence intervals in gray. Significance is defined as *q* < 0.1 (10% FDR). (**E**) Heatmap displaying median *z* scores for inflammatory markers in each of the five consensus clusters for 249 individuals with T21. Rows are organized by hierarchical clustering and split into seven subgroups for clarity. (**F**) Sina plots displaying distributions of DS IFN scores and indicated inflammatory markers across the five immune subtypes identified in (E). Asterisks indicate *q* < 0.1 (10% FDR) for Mann-Whitney tests for each cluster against cluster 1. Boxes in sina plots represent interquartile ranges and medians, with notches approximating 95% confidence intervals. See also fig. S3. n.s., not significant.

Next, we defined correlations between DS IFN scores and 54 inflammatory markers measured by multiplexed immunoassays using the MSD platform, allowing us to examine changes in various cytokines, chemokines, and immune factors with respect to T21 status and correlation with DS IFN scores (Fig. 2, A and B; fig. S3, A and B; and data S3). Among the four IFNs measured on this platform, IFNG (type II) and IFNL1 (type III) displayed significant positive correlations with DS IFN scores, but this was not the case for the type I IFNs, IFNA2 and IFNB1 (Fig. 2, A to D, and fig. S3C). Thus, although the whole blood transcriptome analysis reveals up-regulation of the two type I *IFNRs* encoded on chr21 (*IFNAR1, IFNAR2*; Fig. 1B and fig. S2C) and despite the well-demonstrated hypersensitivity of immune and nonimmune cells from individuals with DS to type I IFN stimulation (*9*, *10*, *12*, *13*, *15*), the DS IFN transcriptional response in the peripheral immune compartment is more strongly associated with the levels of type II and type III IFNs. The overall pattern of correlations is reproduced when using the DS IFN scores composed of 52 and 138 ISGs, with IFNG and IFNL1 consistently ranking at the top of the correlations (fig. S3, A and B). Notably, neither IFNG nor IFNL1 are significantly elevated in the bloodstream of people with DS (Figs. 1H and 2D, fig. S3C, and data S1D). To investigate this phenomenon further, we compared correlations between the DS IFN scores versus validated IFN measurements obtained using the MSD and SOMAscan platforms in DS versus COVID-19 (Fig. 2C) (*22*). This exercise revealed that, whereas the DS IFN score tracks preferentially with IFNG and IFNL1 in DS, this same ISG signature correlates significantly with eight different IFNs in COVID-19, five of which are type I IFNs (i.e., IFNA2, IFNA7/17/21, IFNA1, IFNA4/16, and IFNA10) (Fig. 2C). When analyzing the global transcriptome signatures associated with the variable expression of all 6 IFNRs and the 11 IFNs, once again, IFNG and IFNL1 stood out as having the strongest associations with IFN gamma and alpha signatures (fig. S3, D and E). While expression of all four IFNRs encoded on chr21 also associated with IFN signatures and other inflammatory pathways, the associations were weaker relative to the IFN proteins (fig. S3, D and E). Notably, of the two IFNRs encoded elsewhere in the genome (*IFNGR1* and *IL-10RA*), *IL-10RA* showed the opposite behavior, which could be explained by the fact that it also functions as a receptor subunit for IL-10, a cytokine with anti-inflammatory properties known to antagonize IFN signaling in some settings (*23*). Thus, unlike viral infections involving elevation of multiple IFNs and monogenic type I interferonopathies associated mostly with overproduction of type I IFNs (*18*, *24*), the interferonopathy of DS is more likely associated, at least in the context of the peripheral immune system, with the levels of type II and type III IFNs and driven not by elevated levels of these cytokines but rather by increased expression of their receptors. Overexpression of IFNRs on the surface of immune cells with T21 has been well documented (*10*, *13*).

As expected, the transcriptional DS IFN scores correlated significantly with plasma levels of IP10 (IFN-inducible protein 10; CXCL10), a protein encoded by one of the ISGs in the DS IFN score (Fig. 2, A, B, and D). The third strongest correlation in this analysis was to TNFα, a potent inflammatory cytokine that has been consistently observed to be elevated in DS (Fig. 2, A, B, and D) (*11*, *25*). Elevated TNFα has been implicated in the etiology of several autoimmune and neurological conditions more common in DS (*26*), and its association with DS IFN scores suggests that its elevation in DS could be understood as part of the interferonopathy. Also strongly correlated with the DS IFN scores is IL-6, another key proinflammatory cytokine consistently elevated in DS (Fig. 2, A, B, and D) (*11*, *25*). IL-6 is a potent driver of liver inflammation, inducing production of acute phase proteins such as C-reactive protein (CRP) and serum amyloid A protein (SAA) (*27*), which are also elevated in DS and positively correlated with DS IFN scores (Fig. 2, A, B, and D, and fig. S3C). Notably, many important cytokines elevated in those with T21 do not show significant correlations with the DS IFN scores. An example is thymic stromal lymphopoietin (TSLP), a factor that promotes T helper cell (T_H_2) responses associated with various inflammatory diseases, including allergic inflammation, asthma, and chronic obstructive pulmonary disease (Fig. 2, A, B, and D) (*28*). These results indicate that some, but not all, immune dysregulation observed in DS is associated with the degree of IFN hyperactivity. To investigate this phenomenon further through an orthogonal approach, we performed consensus clustering of all participants with DS based on their inflammatory markers to identify potential subtypes, revealing five major immune profiles in our DS cohort, with cluster 1 showing significantly elevated IFN scores relative to all other clusters (Fig. 2, E and F; see Materials and Methods). In agreement with the Spearman correlation analysis, cluster 1 displays significantly elevated levels of IFNG and multiple components of the IL-6–CRP–SAA pathway, as well as higher levels of macrophage inflammatory protein 1α (MIP1α) and MIP3α (Fig. 2F and fig. S3F). In contrast, clusters 4 and 5, with generally lower IFN scores, display higher levels of TSLP and/or vascular endothelial growth factor C (VEGFC) (Fig. 2, E and F). Thus, IFN hyperactivity is clearly associated with a distinct proinflammatory subtype of DS. Overall, these results further reinforce the notion of a mixed-type interferonopathy in DS, with key contributions of type II and type III IFN signaling, while also demonstrating that IFN hyperactivity is linked to induction of specific major inflammatory pathways dysregulated in DS, such as TNFα and IL-6 signaling.

### IFN hyperactivity accompanies global proteomic changes in DS

**Fig. 3.**
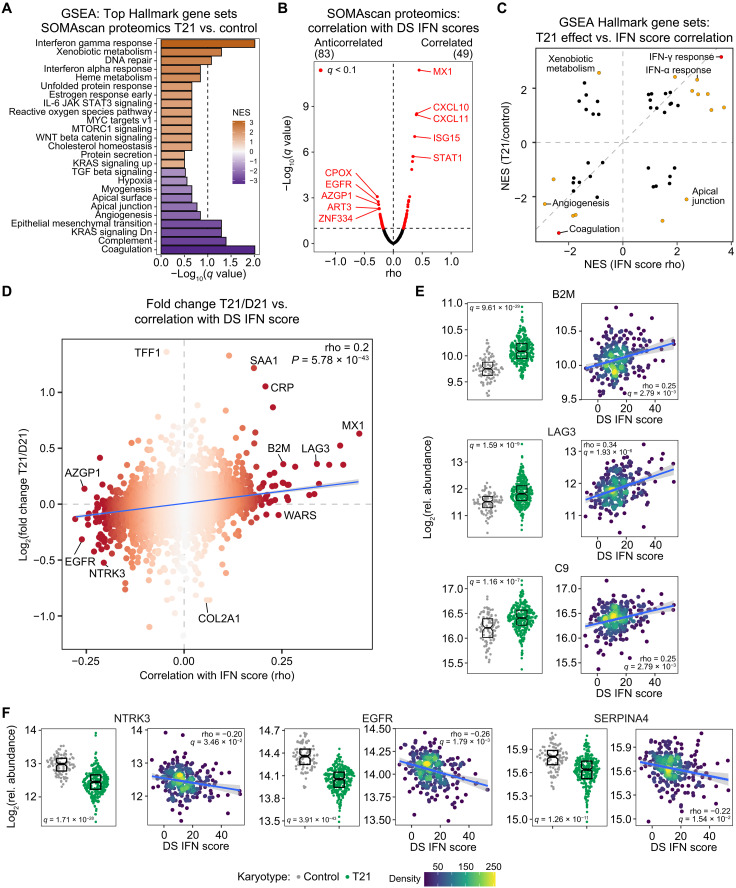
IFN hyperactivity correlates with global proteomics changes in DS. (**A**) Barplot of the top 25 Hallmark gene sets sorted by absolute NES from GSEA of protein changes in the plasma of individuals with T21 (*n* = 316) versus euploid controls (*n* = 103). Bar color represents NES values; bar length represents −log_10_(*q* value). (**B**). Volcano plot displaying the correlations between the transcriptional DS IFN score and all proteins measured in plasma samples with the SOMAscan platform. (**C**) Scatterplot comparing GSEA NES values for protein signatures dysregulated in the proteome of individuals with T21 relative to euploid controls (*y* axis) versus those associated with the transcriptional DS IFN score (*x* axis). Points are colored according to significant enrichment in either (orange) or both (red) analyses. (**D**) Scatterplot comparing the effects of T21 on plasma protein levels (*y* axis) versus their correlation to transcriptional DS IFN scores in 304 individuals with T21 (*x* axis). Color intensity of points increases with absolute rho value. The blue line represents a linear fit through the data, with 95% confidence interval in gray, with Spearman rho and *P* value for the relationship in the top right. (**E** and **F**) Sina plots (left) displaying the effects of T21 on levels of plasma proteins and scatterplots (right) displaying their relationship with transcriptional DS IFN scores among those with T21 (with rho and *q* values for Spearman correlation). Points are colored by density; blue lines represent linear model fit, with 95% confidence intervals in gray. Significance is defined as *q* < 0.1 (10% FDR). Boxes in sina plots represent interquartile ranges and medians, with notches approximating 95% confidence intervals. See also fig. S4.

Next, we investigated the relationship between DS IFN scores and the plasma proteomic changes identified by SOMAscan technology in those with T21. As for the transcriptome analysis, GSEA revealed proteomic signatures indicative of activation of IFN gamma and IFN alpha responses in DS (Fig. 3A and data S4A). Whereas many of the gene sets identified by the proteomics analysis as dysregulated in DS were similar to those identified in the transcriptome analysis (e.g., IFN responses, heme metabolism, and estrogen response) (Fig. 1A), others were not (e.g., down-regulation of the coagulation cascade in the proteomics dataset), thus highlighting the value of proteomics analyses as a complement to transcriptome analyses. Next, to define associations between the strength of IFN hyperactivation and specific proteomic changes, we calculated Spearman correlation scores for all plasma proteins measured versus the DS IFN scores calculated from the whole blood transcriptome analysis (Fig. 3B and data S4B). The whole blood transcriptome–based DS IFN score showed positive correlations with plasma proteins encoded by ISGs (e.g., MX1, CXCL11, CXCL10, ISG15, and STAT1) (Fig. 3B). Again, the overall pattern of correlations was preserved when using DS IFN scores with greater numbers of ISGs (fig. S4, A and B). Comparison of GSEA results revealed that many, but not all, of the same pathways affected by T21 are significantly enriched among the positive (e.g., IFN gamma and alpha responses) and negative (e.g., angiogenesis and coagulation) correlations with the DS IFN scores (Fig. 3C and data S4C).

To further examine the global relationship between the impacts of T21 status versus varying IFN signaling, we compared the fold change in T21/euploid control samples versus the correlation to the RNA-based DS IFN score for all proteins, which demonstrated a significant positive correlation between the two values (Spearman rho 0.2, *P* = 5.78 × 10^−43^; Fig. 3D). Thus, the degree of IFN hyperactivity observed in the transcriptome of individuals with DS correlates with the extent of proteomic changes in their peripheral blood. This exercise revealed different sets of proteins based on their concordant or discordant behavior in relation to T21 status versus IFN signaling (Fig. 3, D and E). A subset of proteins is elevated in T21 but do not show significant associations with IFN scores. This group contains many proteins encoded on chr21 whose elevation could be explained by mere increased gene dosage without obvious links to IFN signaling, such as the transcription factor TFF1 and the collagen family member COL18A1 (fig. S4C). However, this group also contains proteins encoded elsewhere in the genome whose elevation would likely be due to IFN-independent downstream effects of T21, such as MMP1 (matrix metalloproteinase 1) (fig. S4C). Another set of proteins are both elevated in T21 and significantly positively associated with DS IFN scores. This group is highly enriched for immune regulatory factors, including proteins encoded by ISGs in the DS IFN score (e.g., CXCL10), other ISGs not in the DS IFN score (e.g., CXCL11 and GBP1), general markers of inflammation (e.g., the acute phase proteins CRP and SAA1), proteins involved in antigen presentation (e.g., B2M), T cell function (e.g., LAG3), and monocyte/macrophage activation (e.g., CD163) (Fig. 3E and fig. S4D). This class also contains several subunits of the interconnected complement and coagulation cascades, such as C9 (Fig. 3E). A minor class consists of proteins depleted in T21 and positively correlated with IFN scores (e.g., tryptophanyl-tRNA synthetase, WARS; fig. S4E). Another set of proteins are depleted in T21 and not significantly correlated with IFN scores. This subset represents proteins whose abundance in plasma is likely depleted in T21 via IFN-independent mechanisms. This group is enriched for proteins involved in the extracellular matrix organization, cell-cell adhesion, and cell migration, such as the collagen subunit COL2A1 and the proteoglycan PRG3 (fig. S4F). This class also includes several factors involved in platelet function, activation, and degranulation, such as PDGFB, PPBP, and ANGPT1 (fig. S4F and data S4A). Notably, another group of proteins are depleted in T21 and significantly anticorrelated with IFN scores, including several prominent signaling factors involved in growth, development, and morphogenesis, such as the epidermal growth factor receptor (EGFR), the neurotrophic tyrosine kinase receptor 3 (NTRK3), the morphogenic factor and TGFβ antagonist Noggin (NOG), and the Notch ligand Contactin 1 (CNTN1) (Fig. 3F and fig. S4G). This class also contains many important regulators of coagulation and fibrinolysis, such as Kallistatin (SERPINA4), PROC, and SERPINF2 (Fig. 3F and data S4A). Last, a small set contains proteins up-regulated in T21 and negatively correlated with IFN scores, such as AZGP1 (fig. S4H).

Together, these results indicate that IFN hyperactivity is significantly associated with the global proteomic changes caused by T21, whereby individuals with not only the strongest interferonopathy show many changes indicative of inflammation but also the strongest dysregulation of key growth factor signaling pathways, morphogenic factors, and coagulation and fibrinolysis pathways. Furthermore, this analysis points to the likely existence of IFN-dependent versus IFN-independent proteomic signatures in those with T21.

### ISG expression stratifies the degree of immune remodeling in DS

**Fig. 4.**
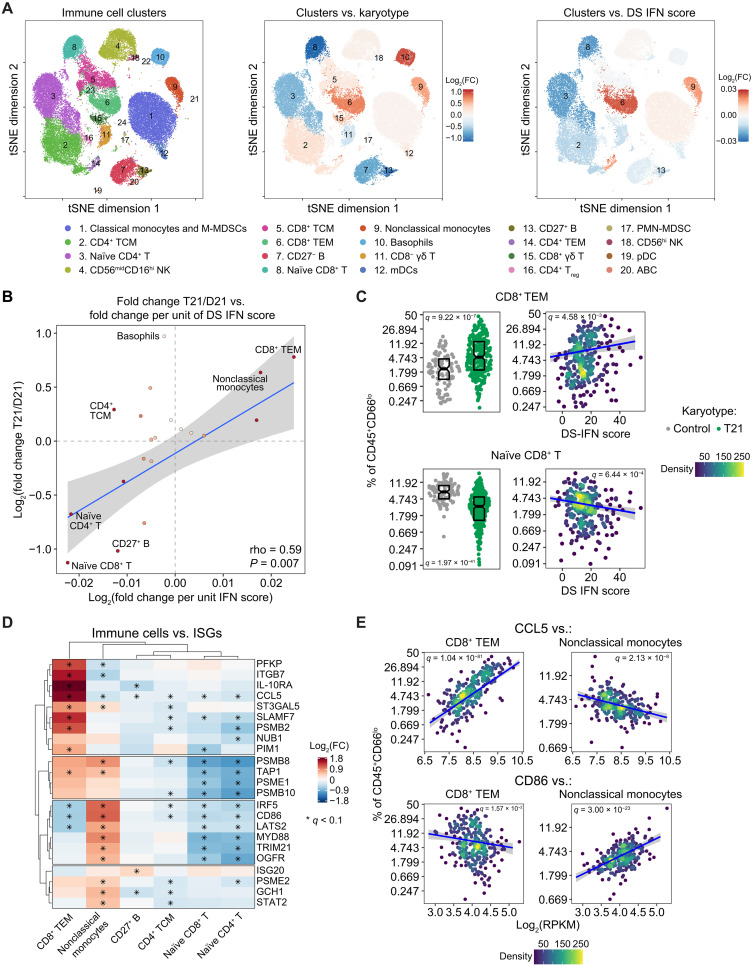
IFN hyperactivity shapes the immune cell landscape in DS. (**A**) *t*-Distributed stochastic neighbor embedding (tSNE) plots of mass cytometry data from 292 individuals with T21, color coded by 20 major immune clusters identified using FlowSOM (left), by fold change (FC) in relative frequency (as percentage of CD45^+^CD66^lo^ cells) in individuals with T21 versus euploid controls (D21) (center), and by fold change in relative frequency per unit DS IFN score (right). A total of 10,000 cells per sample were used for clustering and quantification; 500 cells per sample were displayed. (**B**) Scatterplot comparing the effects of T21 on relative frequencies of immune cell clusters (*y* axis) to their associations with transcriptional DS IFN scores in 277 individuals with T21 (*x* axis). Color intensity of points increases with absolute rho value; the blue line represents a linear fit, with 95% confidence interval in gray. (**C**) Sina plots (left) displaying the effects of T21 on immune cell frequencies and scatterplots (right) displaying relationships to DS IFN scores among those with T21. (**D**) Heatmap displaying log_2_-transformed fold change values (per unit of mRNA expression) for ISGs with ≥1 significant association with immune cell clusters. Asterisks denote *q* < 0.1 by beta regression. Rows and columns are organized by hierarchical clustering, and rows are split into four subgroups for clarity. (**E**) Scatterplots displaying the relationship between relative frequencies of immune cell clusters and whole blood mRNA levels for indicated ISGs among those with T21. Points in scatterplots in (C) and (E) were colored by density, with blue lines representing beta regression fits and 95% confidence intervals in gray; significance is defined as *q* < 0.1 (10% FDR) by beta regression. Boxes in sina plots represent interquartile ranges and medians, with notches approximating 95% confidence intervals. See also figs. S5 and S6.

Next, we investigated associations between DS IFN scores and the immune cell landscape as measured by mass cytometry. People with DS exhibit significant differences in relative frequencies of 14 of the 20 main immune clusters identified by unsupervised FlowSOM clustering of the mass cytometry data (Fig. 4A; figs. S1F and S5, A to C; and data S1F). These differences affect multiple major immune cell lineages, as evidenced by increases in the proportion of basophils, increases in the proportion of differentiated CD4^+^ and CD8^+^ T cell subsets accompanied by decreases in their respective naïve subsets, depletion of B cells, and shifts in the myeloid lineage toward inflammatory subtypes [e.g., nonclassical monocytes and CD1c^+^ myeloid dendritic cells (mDCs)]. To define how these changes correlate to the extent of IFN hyperactivity among individuals with T21, we used beta regression modeling with adjustment for age and sex to test for significant relationships between the DS IFN score and relative frequencies for each immune cell cluster in T21 samples (Fig. 4A, fig. S6A, and data S5A). Results were nearly identical when using 18, 52, or 138 ISGs to calculate DS IFN scores (fig. S6A). Overall, IFN hyperactivity correlated positively with the degree of global immune remodeling in T21 (Spearman rho = 0.59, *P* = 0.007; Fig. 4B). For example, effector memory CD8^+^ T cells (CD8^+^ TEM; cluster 6), which are elevated in T21, show significant positive correlations with the DS IFN score (Fig. 4C). Conversely, naïve CD8^+^ T cells (cluster 8) and naïve CD4^+^ T cells (cluster 3), both of which are depleted in T21, are negatively correlated with the DS IFN score (Fig. 4C and fig. S6B). Nonclassical monocytes (cluster 9), which are elevated in DS, also correlate positively with the IFN score (fig. S6B). CD27^+^ B cells (cluster 13), which are depleted in T21, correlate negatively with the DS IFN score (fig. S6B). Notable deviations from this trend are central memory CD4^+^ T cells (CD4^+^ TCM; cluster 2), which are more frequent in T21 but negatively correlated with DS IFN scores (Fig. S6B).

Next, we investigated whether variations in immune cell frequencies are associated with mRNA expression of specific ISGs in DS. Toward this end, we defined correlations among all 138 non-chr21 ISGs elevated in DS and all 20 immune cell clusters using beta regression modeling (data S5B). This exercise revealed distinct relationships between ISG expression and immune remodeling (Fig. 4D). For example, the ISGs *CCL5* (Rantes) and *IL10RA* (IL-10 receptor subunit A) are the most strongly associated with increased frequencies of CD8^+^ TEMs, but not with increased frequencies of nonclassical monocytes, which are instead associated with overexpression of the costimulatory molecule *CD86* and the transcription factor *IRF5* (Fig. 4, D and E, and fig. S6C). Depletion of naïve CD8^+^ and CD4^+^ T cells is strongly correlated with overexpression of IFN-inducible immunoproteasome subunits such as *PSMB8* and *PSMB10* (Fig. 4, D and E, and fig. S6C), whereas depletion of CD27^+^ B cells was more strongly tied to overexpression of *IL10RA* and *CCL5* (Fig. 4D). Therefore, specific immune changes observed in DS can be linked to different aspects of the IFN transcriptional response and distinct subsets of ISGs.

Together, these results indicate that the degree of IFN hyperactivity is associated with the extent of global immune remodeling in DS, whereby individuals with the most elevated IFN signaling show the most notable differences in immune cell subsets, including the strongest signs of T cell differentiation, B cell depletion, and monocyte activation.

### IFN hyperactivity reveals signs of metabolic cross-talk among immune cell lineages

**Fig. 5.**
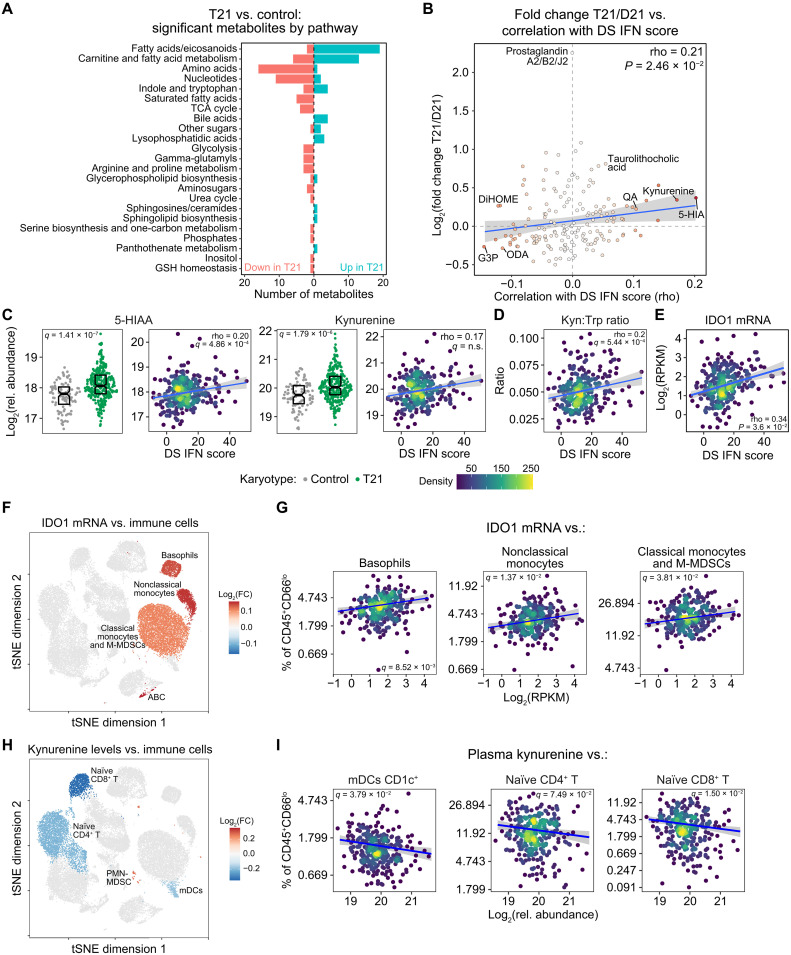
IFN hyperactivity reveals signs of metabolic cross-talk among immune cell lineages. (**A**) Barplot summarizing metabolites with significant differences in relative abundance in plasma of individuals with T21 versus euploid controls (*q* < 0.1, 10% FDR). (**B**) Scatterplot comparing the effects of T21 on plasma metabolites with their correlations to transcriptional DS IFN scores in 304 individuals with T21. The color intensity of points increases with absolute rho value; the blue line represents a linear fit, with 95% confidence interval in gray. (**C** to **E**) Sina plots (left) displaying plasma metabolite levels, kynurenine:tryptophan ratios, or IDO1 mRNA expression and scatterplots (right) displaying their relationships to DS IFN scores in T21. Scatterplots show Spearman correlation rho and *q* values, with significance defined as *q* < 0.1 (10% FDR); points were colored by density, with blue lines representing linear fits and 95% confidence intervals in gray. (**F**) tSNE plot of mass cytometry data for 277 individuals with T21, colored by fold change (percentage of CD45^+^CD66^lo^ cells per unit of IDO1 mRNA expression) for clusters with significant associations with IDO1 mRNA expression (beta regression *q* < 0.1). (**G**) Scatterplots comparing relative cluster frequencies with IDO1 mRNA expression among those with T21. (**H**) tSNE plot of mass cytometry data for 292 individuals with T21, colored by fold change (percentage of CD45^+^CD66^lo^ cells per unit of kynurenine abundance) for clusters with significant associations with plasma kynurenine levels (beta regression *q* < 0.1). (**I**) Scatterplots comparing relative cluster frequencies with plasma kynurenine levels among those with T21. Boxes in sina plots represent interquartile ranges and medians, with notches approximating 95% confidence intervals. For scatterplots in (G) and (I), points were colored by density, with blue lines representing beta regression fits and 95% confidence intervals in gray; significance is defined as *q* < 0.1 by beta regression. See also fig. S7.

Next, we aimed to define associations between DS IFN scores and metabolic changes observed in those with T21. Targeted metabolomics analysis using mass spectrometry methods revealed differential abundance of 119 of the 174 metabolites measured (54 up-regulated and 65 down-regulated), demonstrating the profound impact of T21 on human physiology (fig. S1E and data S1E). Key findings include dysregulation of fatty acid, eicosanoid, and carnitine metabolism; depletion of many circulating amino acids and nucleotides; and activation of the kynurenine pathway of tryptophan catabolism (Fig. 5A). We then defined correlations between DS IFN scores and metabolite levels, which revealed an overall significant positive association between IFN hyperactivity and metabolic dysregulation in DS (Spearman rho 0.21, *P* = 2.46 × 10^−2^; Fig. 5B and data S6). Similar results were obtained using the 52 and 138 ISG scores (fig. S7A). Notably, three of the metabolites most positively associated with DS IFN scores belong to the kynurenine pathway of tryptophan catabolism (i.e., 5HIAA, kynurenine, and quinolinic acid), all of which are elevated in DS (Fig. 5, B and C). Furthermore, the DS IFN scores correlate positively with the kynurenine/tryptophan ratio, a common metric of activation of the kynurenine pathway (Fig. 5D). This result could be explained by IFN-dependent induction of the indoleamine 2,3-dioxygenase 1 (IDO1) enzyme in cells with T21 (*15*), as IDO1 catalyzes the rate-limiting step in the kynurenine pathway (*29*). DS IFN scores correlated positively and significantly with levels of *IDO1* mRNA in individuals with T21 (Fig. 5E).

Activation of the kynurenine pathway via induction of IDO1 is recognized as an immune modulatory mechanism, whereby kynurenine can act as an immunometabolite to suppress the function of effector T cells and NK (natural killer) cells through various mechanisms (*30*). To investigate this pathway in more detail in the context of DS, we tested for associations between *IDO1* mRNA expression or kynurenine levels and relative frequencies of immune clusters using beta regression. Notably, *IDO1* expression displayed significant positive associations with several myeloid subsets, including basophils, nonclassical monocytes, and classical monocytes/monocytic myeloid-derived suppressor cells (M-MDSCs) (Fig. 5, F and G, and fig. S7B). Whereas the association with basophils is unexpected, the association with monocytic lineages agrees with the notion that *IDO1* is expressed preferentially by professional antigen-presenting cells (*30*). The pattern of correlations between kynurenine levels and immune cell subsets was clearly different than that observed for *IDO1*, showing significant negative associations with naïve T cells (CD8^+^ and CD4^+^) and mDCs but a positive association with polymorphonuclear (PMN) MDSCs (Fig. 5, H and I, and fig. S7B). The negative correlation between kynurenine and naïve CD4^+^ T cell subsets could be potentially explained by the fact that kynurenine drives CD4^+^ T cell differentiation toward T regulatory cells (T_regs_) (*30*), yet T_reg_ frequencies were not significantly associated with kynurenine levels in our analysis (data S6C). These findings could be interpreted as a cross-lineage metabolic cross-talk, whereby up-regulation of a metabolic gene in one lineage (i.e., *IDO1* in selected myeloid cell types) leads to changes in cell function in a different lineage through the action of a bioactive metabolite (i.e., kynurenine modulation of T cell function). Together, these results reveal the metabolic signature of IFN hyperactivity signaling in DS, while demonstrating the power of the multiomics datasets to illuminate pathways dysregulated in those with T21.

### IFN hyperactivity distinguishes a clinical subgroup in DS

**Fig. 6.**
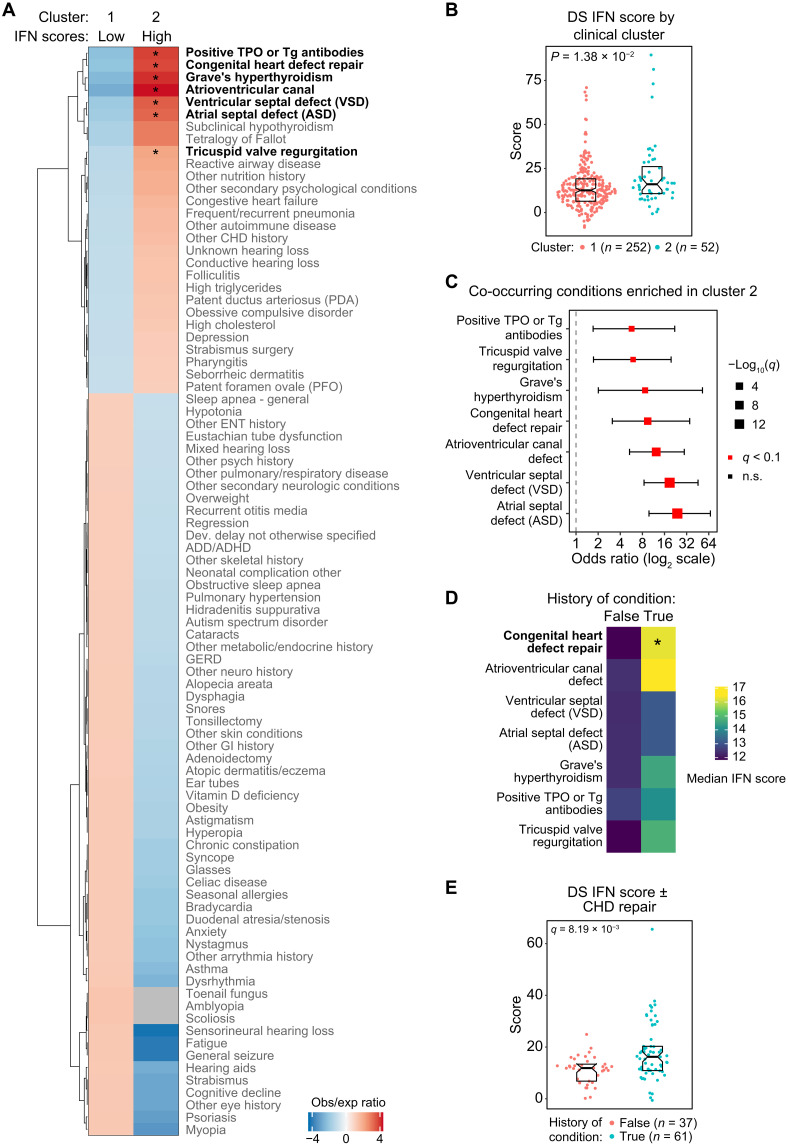
IFN hyperactivity distinguishes a clinical cluster in DS. (**A**) Heatmap comparing the observed over expected ratio for proportions of cases for co-occurring conditions across two clusters of individuals with DS. Rows are organized by hierarchical clustering. Bold row labels indicate *q* < 0.1 (10% FDR) for Fisher’s exact test. (**B**) Sina plot comparing DS IFN score distributions across the two clusters, with *P* value for Mann-Whitney *U* test in the top left. (**C**) Odds ratio plot for Fisher’s exact test of proportions (cases versus controls) across clusters for history of co-occurring conditions. Only conditions with *q* < 0.1 (10% FDR) are displayed. The size of square points is inversely proportional to *q* value; error bars represent 95% confidence intervals. (**D**) Heatmap displaying median DS IFN scores for cases (true) versus controls (false) for each co-occurring condition in (C). (**E**) Sina plot comparing DS IFN score distributions in cases (true) versus controls (false) for “congenital heart defect repair,” with the *q* value for Mann-Whitney *U* test in the top left.

Next, we investigated the interplay between DS IFN scores and common co-occurring conditions in DS. Individuals with DS display elevated prevalence of many medical conditions across the life span, with strong interindividual variability and highly combinatorial occurrence (*5*). To explore the relationship between the patterns of co-occurring conditions and IFN hyperactivity, we calculated pairwise distances between 304 participants with T21, using the Gower method to incorporate information on both history of co-occurring conditions and IFN scores, followed by clustering using the partitioning around medoids (PAM) algorithm (see Materials and Methods). We used only co-occurring conditions that affected at least 10 participants in the T21 cohort, arriving at a two-cluster solution (Fig. 6A), with cluster 2 displaying a significant tendency toward higher DS IFN scores (Fig. 6B). These two clusters of participants display clear differences in their pattern of co-occurring conditions, with seven of these conditions being significantly overrepresented in cluster 2 (Fig. 6, A and C). Notably, the conditions enriched in cluster 2 include multiple forms of CHD (i.e., atrioventricular canal defect, atrial septal defect, ventricular septal defect, and tricuspid valve regurgitation), leading to overrepresentation of individuals with “congenital heart repair” in this group. Also overrepresented within cluster 2 are cases positive for anti–thyroid peroxidase (TPO)/thyroglobulin (TG) antibodies and Grave’s hyperthyroidism. Cluster 2 also shows trends toward increased rates of “subclinical hypothyroidism” (Fig. 6A). Given that cluster 2 showed significant tendency toward higher DS IFN scores (Fig. 6B), we next compared DS IFN scores in those participants with DS with versus without each of the independent conditions overrepresented in this cluster. Affected individuals tend to have higher DS IFN scores, although only reaching statistical significance for the overall category “congenital heart defect repair” (Fig. 6, D and E). Overall, these analyses suggest that individuals with stronger IFN hyperactivity may be more prone to several major comorbidities, although larger numbers of cases/controls for each co-occurring condition would be required for a definitive analysis.

### JAK inhibition attenuates IFN signaling in DS with therapeutic benefit

**Fig. 7.**
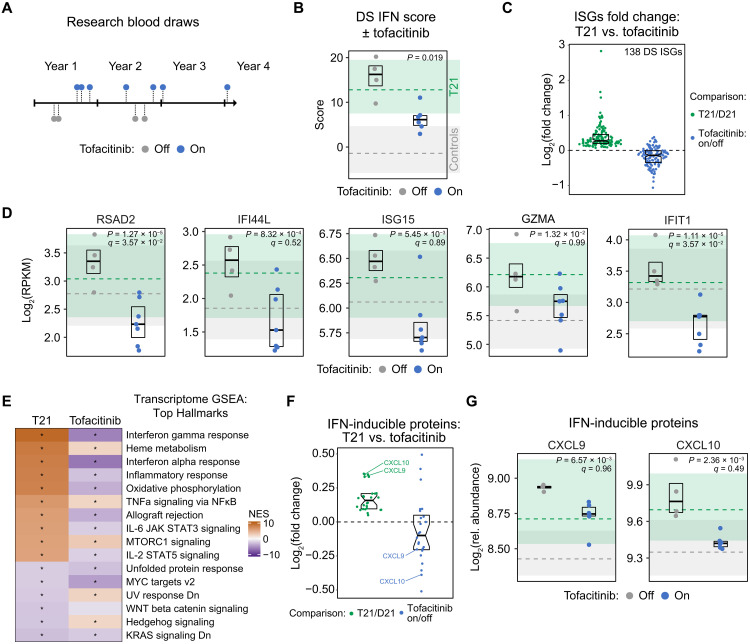
JAK inhibition attenuates IFN signaling with therapeutic benefit in DS. (**A**) Schematic outlining the research blood draw collection schedule and tofacitinib treatment status for the research participant. (**B**) Dot plot comparing DS IFN scores for samples taken when the research participant was off (*n* = 4) versus on (*n* = 7) tofacitinib treatment. Boxes represent interquartile ranges and medians for each group. Shaded areas represent the interquartile ranges for euploid controls (gray) and T21 samples (green) in the HTP cohort, with dashed lines indicating median values. (**C**) Sina plot comparing distributions of fold changes for ISGs in T21/controls (green) versus on/off tofacitinib (blue). Boxes represent interquartile ranges and medians, with notches approximating 95% confidence intervals. (**D**) Dot plots comparing mRNA expression levels for indicated genes in samples taken when the participant was off versus on tofacitinib treatment. Boxes represent interquartile ranges and medians for each group. Shaded areas represent the interquartile ranges for euploid controls (gray) and T21 samples (green), with dashed lines indicating median values. (**E**) Heatmap comparing results of GSEA with Hallmark gene sets for whole blood transcriptome changes in T21/control versus on/off tofacitinib. Color coding represents NES; asterisks indicate gene sets with significant positive (orange) or negative (purple) enrichment (*q* < 0.1, 10% FDR). (**F**) Sina plot comparing the fold change in the plasma levels of 26 IFN-inducible proteins in T21/controls (green) versus on/off tofacitinib (blue). (**G**) Dot plot comparing relative abundance in plasma for indicated proteins in samples taken when the participant was off versus on tofacitinib treatment. Boxes represent interquartile ranges and medians for each group. Shaded areas represent the interquartile ranges for euploid controls (gray) and T21 samples (green), with dashed lines indicating median values. See also fig. S8.

All three types of IFN signaling use JAK1 for signal transduction, suggesting that small molecule inhibitors of this enzyme could ameliorate the interferonopathy of DS (*31*). To test this notion, we evaluated IFN signaling in a research participant taking the JAK inhibitor tofacitinib (Xeljanz) for the treatment of alopecia areata, an autoimmune condition leading to hair loss that is more common in people with DS. This participant has also been affected by other common co-occurring conditions in DS, such as CHD (both ASD and VSD repaired surgically), pediatric pulmonary hypertension, pediatric hypercholesterolemia, AITD, hidradenitis suppurativa, polycystic ovary syndrome, and mild hearing loss. This individual experienced remarkable therapeutic benefit when using tofacitinib for alopecia areata (*32*), leading to periods of voluntary treatment interruption. Over the course of ~3 years, this participant provided blood samples while taking the drug (*n* = 7) and during periods of no treatment (*n* = 4) (Fig. 7A; see Materials and Methods). Using the whole-blood RNA-seq analysis of these samples, we monitored the impact of JAK inhibition on the DS IFN score, which revealed a significant reduction while on the drug (Fig. 7B and data S7A). When not taking tofacitinib, DS IFN scores for this participant fell mostly within the upper range of that observed for individuals with DS. When taking the medicine, the IFN scores decreased toward the high upper range observed for the euploid controls, indicating that therapeutic benefit was achieved without suppression of IFN signaling below the normal range in the general population. Multiple ISGs in the DS IFN score were clearly decreased when the participant was on the drug (e.g., *RSAD2*, *IFI44L*, *ISG15*, and *GZMA*) (Fig. 7, C and D). Broader analysis of all 138 ISGs not encoded on chr21 showed widespread down-regulation while the participant was on the medicine (e.g., *IFIT1*, *MX1*, *OAS3*, and *STAT1*) (Fig. 7, C and D, and fig. S8A). Notably, tofacitinib treatment led to a modest increase in mRNA expression of the four *IFNRs* encoded on chr21, but otherwise decreased expression of ISGs encoded on chr21, such as *MX1* (fig. S8A). Thus, although the drug does not correct the increased IFNR expression driven by T21, it dampens downstream IFN signaling.

To assess the impact of JAK inhibition more globally, we performed GSEA on the whole blood transcriptome against Hallmark gene sets. Of the top 10 positively enriched gene signatures in T21, 7 were negatively enriched (i.e., down-regulated) by the drug, including IFN gamma response, IFN alpha response, inflammatory response, oxidative phosphorylation, allograft rejection, mammalian target of rapamycin complex 1 (mTORC1) signaling, and IL-2/STAT5 signaling (Fig. 7E and fig. S8B). Thus, JAK inhibition can normalize many of the transcriptome signatures associated with DS in the peripheral immune compartment.

Last, to define whether attenuation of the ISG signature was also observed at the protein level, we evaluated the impact of JAK inhibition on the plasma proteome by evaluating 10 samples obtained from this research participant (6 on treatment and 4 off treatment) (data S7B). The SOMAscan platform used was able to measure levels of 65 of the proteins encoded by the 138 DS ISGs. Of these proteins, 26 were significantly elevated in the plasma of participants with DS in the HTP cohort, many of which were down-regulated by tofacitinib treatment in this research participant (e.g., CXCL9 and CXCL10; Fig. 6, F and G). Together, these results indicate that the interferonopathy of DS is amenable to pharmacological modulation with JAK inhibitors, with potential for multidimensional benefits in this population.

### JAK inhibition attenuates hyperactive IFN signaling across multiple organs

**Fig. 8.**
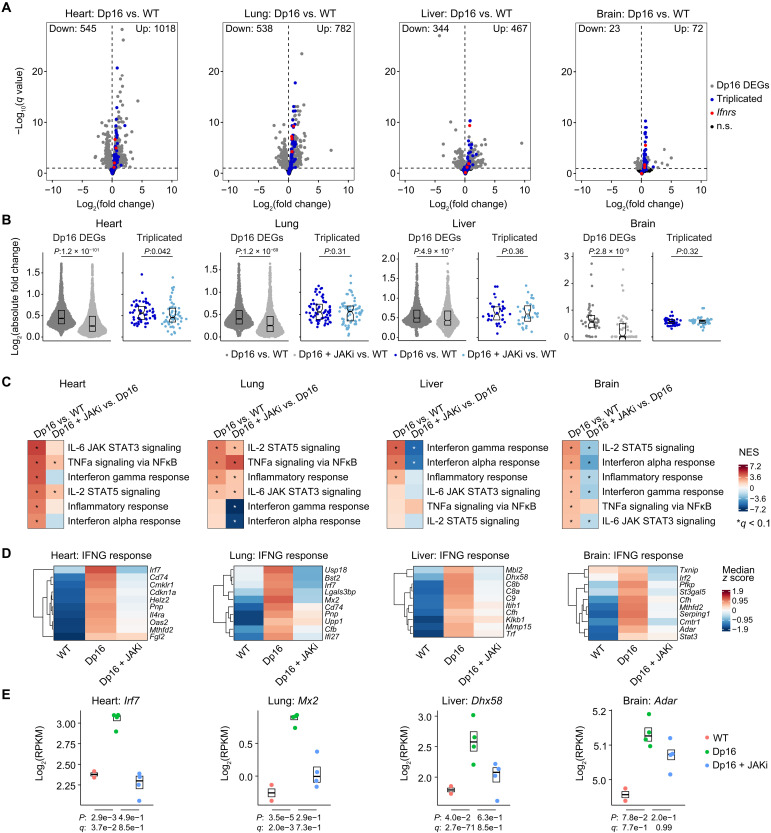
JAK inhibition attenuates global dysregulation of gene expression in a mouse model of DS. (**A**) Volcano plots summarizing results of transcriptome analysis using DESeq2 of indicated tissues obtained from vehicle-treated adult WT versus Dp16 mice, highlighting genes triplicated in Dp16 (blue), the four *Ifnrs* (red), and nontriplicated DEGs (gray), with all other genes in black; *q* values were determined using the Benjamini-Hochberg approach. Number of animals: WT vehicle, *n* = 2 (2 males); Dp16 vehicle, *n* = 4 (2 males and 2 females); and Dp16 + JAKi, *n* = 4 (2 males and 2 females). (**B**) Sina plots displaying absolute fold changes for nontriplicated Dp16 DEGs (gray) and genes triplicated in Dp16 (blue) for Dp16 (vehicle) versus WT or Dp16 + JAKi (baricitinib treatment) versus WT comparisons, with *P* values for two-sided Mann-Whitney *U* tests, boxes representing interquartile ranges and medians, and notches approximating 95% confidence intervals. (**C**) Heatmaps displaying NES from GSEA of transcriptome fold changes for the indicated comparisons for inflammation-related Hallmark gene sets, sorted by NES for Dp16/WT; asterisks indicate *q* < 0.1 defined by GSEA. (**D**) Heatmaps representing median expression *z* scores per genotype (calculated from RPKM values) for example genes from the indicated gene sets. (**E**) Sina plots for example genes from the indicated gene sets; *P* and *q* values were determined by DESeq2 with significance defined as *q* < 0.1.

Last, we investigated IFN signaling and the impacts of JAK inhibition across multiple organ systems in a mouse model of DS. Toward this end, we used Dp(16Lipi-Zbtb21)1Yey/J mice (referred to henceforth as Dp16) (*33*), a well-characterized model that harbors a segmental duplication of a region of murine chr16 orthologous to human chr21, leading to triplication of ~120 genes, including the four *Ifnrs*. Dp16 mice display many phenotypes relevant to DS (*33*), including a dysregulated immune response resulting in lethal hypersensitivity to IFN-inducing agents that can be counteracted with JAK1 inhibitors (*34*). Furthermore, correction of *Ifnr* copy number in this model rescues multiple hallmarks of DS (*17*). We therefore examined the effects of JAK inhibition on gene expression in key organs relevant to DS pathophysiology. Transcriptome analysis of heart, lung, liver, and brain tissues from adult Dp16 mice identified hundreds of differentially expressed genes (DEGs) up-regulated or down-regulated in this strain relative to wild-type (WT) controls (Fig. 8A). Expectedly, most triplicated genes were up-regulated across Dp16 tissues, including the *Ifnrs*. We then analyzed expression changes in adult Dp16 mice treated for several weeks with the JAK1/2 inhibitor baricitinib, which we previously reported rescues the immune hypersensitivity phenotype (*34*). Notably, baricitinib treatment causes a global attenuation of gene expression changes in all tissues (Fig. 8B). Although expression of triplicated genes is largely unaffected by the drug, DEGs encoded elsewhere in the genome display a significant overall decrease in absolute fold changes. GSEA identified many gene signatures dysregulated in Dp16 that were attenuated upon baricitinib treatment (fig. S9A). Consistent with previous work (*34*), GSEA detected up-regulation of multiple inflammatory signatures in all tissues, including IFN alpha and IFN gamma responses (Fig. 8C). Baricitinib attenuated the expression of multiple ISGs in all tissues tested, with variable effects on other inflammatory signatures (Fig. 8, C to E).

Notably, many other gene expression signatures dysregulated in Dp16 were also attenuated by baricitinib treatment (fig. S9A). Salient examples include up-regulation of genes involved in extracellular matrix remodeling and epithelial-to-mesenchymal transition (EMT) in the heart (e.g., *Vegfa*), activation of mTORC1 signaling in the lung (e.g., *Hspa5*), induction of complement and coagulation factors in the liver (e.g., *C8b*), and down-regulation of oxidative phosphorylation genes in the brain (e.g., *Retsat*) (fig. S9, B and C). Overall, these results indicate that JAK inhibition has broad effects not only on signaling pathways dysregulated in DS, most prominently inflammatory signatures, but also on many other pathways of potential relevance for DS pathophysiology.

## DISCUSSION

The genetic cause of DS has been known since 1959, when Lejeune and colleagues (*1*) demonstrated the presence of an extra copy of chr21 in cells from individuals with DS. Forty years later, at the dawn of the new millennium, chr21 was largely sequenced, leading to the identification of ~220 protein-coding genes on this chromosome (*35*). Since then, the field has been challenged by the difficulty in defining cause-effect relationships between overexpression of specific genes on chr21 and/or general effects of the aneuploidy and the highly heterogeneous developmental and clinical hallmarks of DS. Increasing appreciation for additional research in this area has been driven by the remarkable growth of the population with DS over the last 50 years (*36*) and also by the observation that T21 modulates the appearance and severity of many medical conditions also affecting the general population (*2*). Beyond a core set of developmental and neurological phenotypes, individuals with DS show lower rates of some major medical conditions, such as most solid malignancies and hypertension, while being at high risk of others, such as Alzheimer’s disease, specific autoimmune disorders, CHD, autism, and severe COVID-19 (*5*). Therefore, elucidating the mechanisms by which T21 causes this distinct clinical profile would benefit not only people with DS but also the general population.

Within this context, we report here an integrated multiomics analysis of IFN hyperactivity among individuals with DS. In 1974, Tan and colleagues (*37*) reported that cells with T21 were hypersensitive to early IFN preparations. Thereafter, several reports by the Epstein and Maroun labs further documented hyperactive IFN signaling in human cell cultures and early animal models of DS (*16*, *38*–*44*). However, these pioneering studies did not lead at the time to a widespread appreciation that DS could be understood, in part, as a disorder caused by chronic IFN hyperactivity and consequent immune dysregulation across the life span. Recently, with the advent of omics approaches, it became clear that IFN hyperactivity is a top hallmark of the transcriptome, proteome, metabolome, and immune changes elicited by T21 (*9*–*13*, *15*). Mechanistically, this could be explained in part by the fact that four of the six IFNR subunits are encoded on chr21. In multiple mouse models carrying triplicated *Ifnr* genes, IFN hyperactivity is a conserved biosignature across diverse brain regions and developmental stages (*45*). Furthermore, normalization of *Ifnr* copy number attenuates diverse hallmarks of DS, including embryonic development and neuronal viability (*16*), as well as immune hypersensitivity, septal heart defects, craniofacial abnormalities, and cognitive impairments (*17*). Today, the interferonopathy of DS is a subject of intense study (*46*, *47*), including ongoing clinical trials testing the safety and efficacy of JAK inhibition in DS (NCT04246372 and NCT05662228) (*32*, *48*). Despite these advances, the contribution of chronic IFN hyperactivity to the myriad phenotypes and clinical effects observed in the population with DS remains to be elucidated. Notably, IFN hyperactivity is a recognized driver of type I interferonopathies, a group of monogenic disorders leading to overproduction of IFNs, as well as a range of autoinflammatory conditions, most prominently SLE (*18*, *19*, *49*). Therefore, our integrated multiomics analysis of IFN hyperactivity in DS could potentially provide insights into the pathogenesis of other medical conditions.

Using RNA-based IFN scores, we defined associations between variable IFN signaling and global changes in the proteome, peripheral immune cell lineages, metabolomic changes, and clinical diagnoses in DS. One key conclusion from these efforts is that the interferonopathy of DS should be considered as a “mixed-type interferonopathy” associated with widespread overexpression of type I, type II, and type III IFNRs, with clear potential for type II and type III IFNs to be major contributors to IFN hyperactivity and dysregulation of downstream events. Although multiple immune and nonimmune cells with T21 display hypersensitivity to type I IFNs (*9*, *10*, *13*, *37*), our results demonstrate that the peripheral IFN transcriptional response correlates instead with circulating protein levels of IFNG and IFNL1. Although individuals with DS do not present markedly elevated levels of circulating IFNs as seen in other interferonopathies or during viral infections, the IFN transcriptional response is nonetheless elevated, tracking preferentially with IFNG levels. This observation could have clear therapeutic implications in DS, suggesting that targeting type I IFN specifically, such as in the case of SLE treatment with the monoclonal antibody sifalimumab, may have lesser therapeutic value in DS. Instead, targeting JAK1, which is required for all three types of IFN signaling, could prove more beneficial.

Another key conclusion is that IFN hyperactivity correlates significantly with the global effects of T21 on the circulating proteome, immune cell lineages, and metabolic changes. Consistently, individuals with DS with the highest IFN transcriptional responses show the most pronounced changes in their multiomics profiles. While some of these correlations were perhaps expected, such as the positive association between IFN hyperactivity and various inflammatory markers, others are worthy of further investigation, such as the observed negative correlation between IFN activity and circulating levels of factors involved in major developmental pathways (e.g., EGFR, NTRK3, NOG, and CNTN1). Throughout these analyses, we defined molecular and cellular changes observed in DS that could be associated to IFN hyperactivity versus those that could not. For example, whereas depletion of naïve T cell subsets is clearly associated with elevated IFN signaling, increased frequency of basophils is not. Therefore, the datasets described here provide a resource for the community to dissect different molecular and cellular pathways underlying the pathobiology of DS. In this regard, depletion of naïve T cell subsets concurrent with increase in T cell effector types could be explained by the known effects of IFN signaling on T cell activation and differentiation, and likely driven by overexpression of one or more IFNRs. However, other mechanisms must be invoked at this point to explain the elevated frequencies of basophils in DS. One important caveat is that our analysis is cross-sectional in nature, which prevents an evaluation of changes that could be caused by chronic IFN hyperactivity over long periods of time. Thus, it is possible that an increase in basophils is part of the interferonopathy of DS, but not easily associated with IFN activity at a single point in time.

A key benefit of the multiomics datasets presented here is that they enable delineations of molecular and cellular pathways not easily elucidated otherwise. This is exemplified by our study of the kynurenine pathway. Somewhat expectedly, individuals with the stronger IFN transcriptional responses showed the highest levels of circulating tryptophan catabolites produced within the kynurenine pathway, which could be explained by IFN-driven overinduction of IDO1 (*15*). However, the immune cell types associated with IDO1 expression and kynurenine production are different. Whereas IDO1 expression associates with elevated frequency of monocytes and basophils, kynurenine levels associate with depletion of naïve T cell subsets. This result could be explained by an immune cross-talk between these key myeloid and lymphoid cell types, whereby IDO1 induction in the myeloid lineage would lead to production and secretion of kynurenine, which in turn could exert immunomodulatory effects in T cells and other lymphoid cell types (*30*).

Integrated analysis of IFN scores and clinical metadata revealed that IFN hyperactivity at a single cross-sectional time point associates with increased probability of certain diagnoses, most prominently CHD and AITD. Various forms of CHD affect ~50% of newborns with T21, but the etiology of this hallmark of DS remains unknown. Recently, we demonstrated that normalization of *Ifnr* copy number prevents CHD in the Dp16 mouse model of DS, and that *Ifnr* triplication causes transcriptome changes in the developing murine heart, indicative of activated JAK/STAT signaling and decreased cell proliferation (*17*). Furthermore, single-nucleotide polymorphisms (SNPs) in the human *IFNR* gene cluster have been associated with CHD risk in DS (*50*). These results support the notion that elevated IFN signaling during embryonic development could impair organogenesis (*51*, *52*), while suggesting that individuals with DS who experienced high IFN activity in utero would be more likely to have both a history of CHD and AITD and stronger IFN hyperactivity later in life. The association between IFN hyperactivity and history of AITD in DS is supported by observations in the general population, as SNPs linked to elevated IFN signaling have been associated with increased risk of diverse autoimmune disorders (*53*), and whereby therapeutic administration of recombinant IFNs was shown to increase the risk of AITD (*54*).

One key limitation of our study is that, although we defined informative significant correlations between IFN transcriptional responses and various molecular and cellular events in DS, these associations should be interpreted with caution when trying to infer cause-effect relationships. Nevertheless, the analyses of longitudinal samples collected from an individual undergoing periodic treatments with the JAK inhibitor tofacitinib demonstrated that IFN hyperactivity is attenuated in this individual without overt immune suppression, with consequent normalization of many transcriptional signatures dysregulated in DS, thus involving elevated JAK/STAT signaling as a driver of many of the changes observed. Although the limited sample size obtained from a single individual prevents a definitive assessment of the impacts of JAK inhibition on the pathobiology of DS, these results support the ongoing testing of tofacitinib in larger cohorts, including assessment of safety, efficacy to normalize diverse inflammatory markers, effects on autoimmune skin pathology and AITD, and analyses of effects on neurological health and quality of life (NCT04246372 and NCT05662228).

Results obtained in the Dp16 mouse model of DS illuminate the potential beneficial impacts of JAK inhibition across diverse organ systems. Consistently, baricitinib treatment attenuated global gene expression changes without significantly affecting overexpression of the triplicated genes, indicating that JAK/STAT signaling contributes to a sizable fraction of the global effects of T21, even in the controlled, pathogen-free environment of the vivarium. These results also point to effects of elevated JAK/STAT signaling well beyond inflammatory pathways and immune control. For example, JAK inhibition normalized signatures indicative of extracellular matrix remodeling in the heart (e.g., EMT); signatures of dysregulated mTORC1 signaling in the lung, signs of aberrant activation of the coagulation and complement cascades in the liver; and signatures of decreased mitochondrial metabolism in the brain. Future research studies will be needed to define the potential benefits of such attenuation of gene expression changes in terms of organ development and function.

Recent advances indicate that immune modulation could potentially address diverse aspects of DS across the life span, even perhaps prenatally. Correction of *Ifnr* copy number in mouse models of DS rescued, either partially or totally, key phenotypes that initiate during embryonic development, such as CHD and craniofacial anomalies (*17*). Furthermore, prenatal treatment with the anti-inflammatory and antioxidant natural compound apigenin improved early development and cognitive function in mouse models of DS, concomitant with reduction of IFNG levels (*55*). Although intake of JAK inhibitors is not recommended during pregnancy, tofacitinib use during pregnancy in rheumatic diseases or ulcerative colitis was not associated with increased risk to the fetus relative to risks attributed to the underlying disease (*56*–*58*). After birth, JAK inhibitors could potentially benefit children with DS with signs of strong immune dysregulation, including those affected by any of the approved indications for the general population, such as myelofibrosis, polycythemia vera, rheumatoid arthritis, psoriatic arthritis, juvenile idiopathic arthritis, axial spondyloarthritis, ankylosis spondylitis, ulcerative colitis, atopic dermatitis, alopecia areata, graft-versus-host disease, and COVID-19 (*59*); however, not all of these indications are approved for the pediatric population. Notably, tofacitinib is approved for the treatment of children and adolescents 2 years and older with active polyarticular course juvenile idiopathic arthritis (*59*). Later in life, JAK inhibition may benefit those with a high burden of autoimmune conditions more prevalent in DS, such as AITD, celiac disease, and various immune skin conditions, all of which have been tied in the general population to either IFN hyperactivity or cytokines shown here to associate with IFN scores in DS (*60*, *61*). Individuals with DS are at high risk of severe complications and mortality from SARS-CoV-2 infections (*6*, *62*) and stand to benefit from JAK inhibitors and other anti-inflammatory agents approved for severe COVID-19 (*63*). Last, JAK inhibitors could potentially benefit individuals with signs of immune-related neurological dysfunction, such as in cases of Down syndrome regression disorder (DSRD) (*64*). Tofacitinib is now being tested in a clinical trial for DSRD (NCT05662228). In mouse models of Alzheimer’s disease, IFNRs encoded on human chr21 were found to be required for disease progression (*65*, *66*), and type I IFN signaling was shown to drive neuroinflammation, microglial dysfunction, neuronal senescence, and death in experimental models of DS and Alzheimer’s disease (*67*, *68*). In all these scenarios, we hypothesize that JAK inhibitors may be most beneficial to those individuals displaying high DS-IFN scores or high levels of cytokines associated with the DS-IFN score (e.g., TNFα, IL-6, and IP10), but the value of such a stratification strategy will require additional research. In summary, the results described here advance our collective understanding of both the interferonopathy of DS and the broader impacts of IFN signaling in human biology.

## MATERIALS AND METHODS

### Study design

All study participants were enrolled in the Crnic Institute’s HTP under a study protocol approved by the Colorado Multiple Institutional Review Board (COMIRB 15-2170 and NCT02864108; see also www.trisome.org). Written informed consent was obtained from all study participants or their legal guardians. Biospecimen collection in the HTP biobank includes a blood draw, a tongue swab, and optional urine and/or stool samples. A clinical history for each participant was curated from both medical record review and a participant/family report, with medical record taking precedence in cases of discordance between the two sources. All co-occurring conditions represent a past or current diagnosis of a condition, which may or may not have been active at the time of the blood draw. The biological datasets described here were generated from deidentified blood-derived biospecimens and linked to demographics and clinical metadata for analysis. The overall HTP cohort used for this study consisted of 502 individuals, 356 with DS. Cohort information specific to each dataset can be found in data S1A.

### Blood sample collection and processing

Peripheral blood samples were collected into PAXgene RNA tubes (QIAGEN, catalog no. 762165) and BD Vacutainer K2 EDTA tubes (BD, catalog no. 366643). Samples in PAXgene tubes were processed for RNA-seq as described below. For mass cytometry, 2 × 0.5 ml of whole blood was withdrawn from each EDTA tube for processing as described below. For the remaining measurements, EDTA tubes were processed within 2 hours of blood draw by centrifugation at 700*g* for 15 min to separate plasma, buffy coat (white blood cells), and red blood cells, which in turn were aliquoted, flash-frozen, and stored at −80°C. Subsequent processing was carried out as described below, with aliquots selected to minimize freeze/thaw cycles.

### Whole-blood RNA-seq

Whole-blood RNA was extracted from PAXgene RNA tubes and purified using a PAXgene blood RNA kit (QIAGEN, catalog no. 762164). RNA quality was assessed using a 2200 TapeStation system (Agilent) and quantified on a Qubit fluorometer (Thermo Fisher Scientific). Globin RNA depletion, poly-A(+) RNA enrichment, and strand-specific library preparation were carried out using a GlobinClear kit (Thermo Fisher Scientific, catalog no. AM1980), NEBNext Poly(A) mRNA magnetic isolation module, and NEBNext Ultra II directional RNA library prep kit for Illumina (New England Biolabs, catalog nos. E7490 and E7760). Paired-end 150–base pair (bp) sequencing was carried out on an Illumina NovaSeq 6000 instrument by Novogene Co. Ltd. and delivered in FASTQ format.

### SOMAscan plasma proteomics

A total of 125 μl of EDTA plasma was analyzed by SOMAscan using established protocols (*69*). Each of the 4500+ SOMAmer reagents binds a target peptide and is quantified on a custom Agilent hybridization chip. Normalization and calibration were performed according to SOMAscan Data Standardization and File Specification Technical Note (SSM-020) (*69*). The output of the SOMAscan assay is reported in relative fluorescence units (RFU).

### Profiling of plasma inflammatory markers using MSD assays

For each EDTA plasma sample, two technical replicates (12 to 25 μl depending on required dilution) were measured using the MSD multiplex immunoassay platform V-PLEX Human Biomarker 54-Plex Kit (catalog no. K15248D) on a MESO QuickPlex SQ 120 instrument. Assays were performed according to the manufacturer’s instructions, and concentration values were calculated against a standard curve using the provided calibrators. MSD data are reported as concentration values in picograms per milliliter of plasma.

### Mass cytometry of white blood cells

For mass cytometry analysis, 2 × 0.5 ml of whole blood samples underwent red blood cell lysis and white blood cell fixation using the TFP FixPerm Buffer (Transcription Factor Phospho Buffer Set, BD Biosciences, catalog no. 558049), followed by a wash in 1× phosphate-buffered saline (PBS) (Rockland, catalog no. MB-008). White blood cells were then resuspended in cell staining buffer (CSB; Fluidigm, catalog no. 201068) and stored at −80°C. Before antibody staining, samples were thawed at room temperature, washed in CSB, barcoded using a Cell-ID 20-Plex Pd barcoding kit (Fluidigm, catalog no. PRD023), and combined per batch. Each batch was composed of up to 19 samples along with a common reference sample. Antibodies used for staining were either purchased preconjugated to metal isotopes or conjugated using a Maxpar antibody labeling kit (Fluidigm, catalog no. 201160B). See data S9 for antibody details. Staining dilutions for each antibody were titrated and validated using the common reference sample and comparison to relative frequencies for major cell types obtained by independent flow cytometry analysis. Staining of surface markers was carried out in CSB for 30 min at 4°C, with the addition of an Fc receptor binding inhibitor (eBioscience/Thermo Fisher Scientific, catalog no. 14-9161-73), followed by a wash with CSB. Cells were then permeabilized in buffer III (Transcription Factor Phospho Buffer Set, BD Pharmingen, catalog no. 563239) for 20 min at 4°C and washed with perm/wash buffer (Transcription Factor Phospho Buffer Set, BD Pharmingen, catalog no. 563239). Staining of intracellular transcription factors and phospho-epitopes was carried out in perm/wash buffer (Transcription Factor Phospho Buffer Set, BD Pharmingen, catalog no. 563239) for 1 hour at 4°C, followed by a wash with CSB. Barcoded and stained cells were then labeled with Cell-ID Intercalator-Ir (Fluidigm, catalog no. 201192A) and analyzed on a Helios instrument (Fluidigm). Mass cytometry data were exported as v3.0 FCS files for preprocessing and analysis.

### Mass spectrometry–based plasma metabolomics and lipidomics

Plasma samples were thawed on ice and extracted via a modified Folch method (chloroform/methanol/water 8:4:3). Briefly, 20 μl of sample was diluted in 130 μl of liquid chromatography–mass spectrometry grade water, 600 μl of ice-cold chloroform/methanol (2:1) was added, and the samples were vortexed for 10 s. Samples were then incubated at 4°C for 5 min, quickly vortexed (5 s), and centrifuged at 14,000*g* for 10 min at 4°C. The top (i.e., aqueous) phase was transferred to a new tube for metabolomics analysis and flash-frozen. The bottom (i.e., organic) phase was transferred to a new tube for lipidomics analysis and then dried under N_2_ flow. Analyses were performed using a Vanquish UHPLC coupled online to a Q Exactive high-resolution mass spectrometer (Thermo Fisher Scientific). Samples (10 μl per injection) were randomized and analyzed in positive and negative electrospray ionization modes (separate runs) using a 5-min C18 gradient on a Kinetex C18 column (Phenomenex) as described (*70*). Data were analyzed using Maven in conjunction with the Kyoto Encyclopedia of Genes and Genomes database and an in-house standard library.

### JAK inhibition in the Dp16 mouse model of DS

Experiments were approved by the Institutional Animal Care and Use Committee at the University of Colorado Anschutz Medical Campus under protocol #00111 and performed in accordance with National Institutes of Health (NIH) guidelines. The Dp16 strain has been previously described (*33*). Dp16 mice were originally purchased from the Jackson Laboratory and also gifted by D. Bianchi’s laboratory (NIH) and then intermixed and maintained on the C57BL/6J background in specific pathogen–free conditions. Mice were housed separately by sex in groups of one to five mice per cage under a 14-hour light:10-hour dark cycle with controlled temperature and 35% humidity and had ad libitum access to food (6% fat diet) and water. For genotyping, genomic DNA was prepared from 1 to 2 mm of toe, tail, or ear tissue for automated genotyping by reverse transcription polymerase chain reaction with specific probes designed for each gene (Transnetyx). Mice (7 to 11 weeks old) were randomized into treatment groups and treated with baricitinib (JAKi) (10 mg/kg) or an equivalent volume of 0.5% methylcellulose vehicle once a day via oral gavage for 17 days. Mice were euthanized by CO_2_ asphyxiation and cervical dislocation and then immediately perfused with 1× PBS using a Perfusion Two Automated Perfusion Instrument (Leica). The number of animals is as follows: WT vehicle, *n* = 2 (2 males), Dp16 vehicle, *n* = 4 (2 males and 2 females), and Dp16 JAKi, *n* = 4 (2 males and 2 females). Brain, heart, liver, and lung tissues were homogenized for 30 s using a Mini-Beadbeater-24 (BioSpec Products) and frozen at −80°C. Total RNA was isolated using the AllPrep Kit (QIAGEN) following the manufacturer’s instructions. Library preparation was carried out using a Universal Plus mRNA Kit Poly(A) (Tecan). Paired-end, 150-bp sequencing was carried out on an Illumina NovaSeq 6000. See below for details on RNA-seq analysis.

### Statistical analyses

Data preprocessing, statistical analysis, and plot generation for all datasets were carried out using R (R 4.0.1/RStudio 2022.02.0/Bioconductor 3.11) (*71*, *72*) as detailed below. All figures were assembled in Adobe Illustrator v25.4.5.

#### Data visualization

For comparison of data distributions between different categories/groups, sina plots showing all points jittered horizontally by local density, modified with boxes representing medians and interquartile ranges, were generated using ggplot2 and the geom_sina() function from the ggforce R package. For comparison of continuous data, *XY* scatterplots with points colored by local density were generated using a custom density function and ggplot2. Heatmaps were generated using the ComplexHeatmap and tidyheatmap R packages.

#### Gene set enrichment analysis

GSEA (*73*) was carried out in R using the fgsea package (v 1.14.0), using Hallmark gene sets, and log_2_-transformed fold changes (for RNA-seq), log_2_(fold change) multiplied by −log_10_(*P* value) (for SOMAscan proteomics), or Spearman rho values (for correlations) as the ranking metric.

#### Spearman correlation analysis

Spearman correlation coefficients (rho) and *P* values were calculated for the indicated datasets using the rcorr() function from the Hmisc package (v 4.4-0), with Benjamini-Hochberg adjustment of *P* values and an estimated false discovery rate (FDR) threshold (*q*) of 0.1.

#### Analysis of whole-blood RNA-seq data

RNA-seq data yield was ~33 × 10^6^ to 103 × 10^6^ raw reads and ~21 × 10^6^ to 69 × 10^6^ final mapped reads per sample. Data quality was assessed using FASTQC (v0.11.5) and FastQ Screen (v0.11.0). Trimming and filtering of low-quality reads was performed using bbduk from BBTools (v37.99) and fastq-mcf from ea-utils (v1.05). Alignment to the human reference genome (GRCh38) was carried out using HISAT2 (v2.1.0) in paired, spliced-alignment mode against a GRCh38 index and Gencode v33 basic annotation GTF, and alignments were sorted and filtered for mapping quality (MAPQ > 10) using Samtools (v1.5). Gene-level count data were quantified using HTSeq-count (v0.6.1) with the following options (--stranded=reverse –minaqual=10 –type=exon --mode=intersection-nonempty) using a Gencode v33 GTF annotation file. Differential gene expression in T21 versus D21 was evaluated using DESeq2 (version 1.28.1) (*74*), with *q* < 0.1 (10% FDR) as the threshold for differential expression.

##### DS IFN scores

To capture the degree of IFN signaling in each sample as a single value, we calculated RNA-seq–based DS IFN scores as follows: First, gene-wise *z* scores were calculated from age- and sex-adjusted reads-per-kilobase-per-million (RPKM) values for each sample, based on the mean and SD of the euploid control samples. Second, sample-wise DS IFN scores were calculated as the sum of *z* scores for ISGs, with significant mean fold change of at least 1.5 in the T21 group versus the euploid control group, excluding *IFNAR2*, *MX1*, and *MX2*, which are encoded on chr21. This led to a list of 18 genes shown in fig. S2D.

#### Analysis of SOMAscan proteomics data

Normalized data (RFU) in the SOMAscan adat file format were imported to R using the SomaDataIO R package (v3.1.0). Extreme outliers were classified per karyotype and per analyte as measurements more than three times the interquartile range (IQR) below or above the first and third quartiles, respectively (below Q1 − 3 × IQR or above Q3 + 3 × IQR), and excluded from further analysis. Differential abundance analysis for SOMAscan proteomics was performed using linear regression in R with log_2_-transformed relative abundance as the outcome/dependent variable; T21 status as the predictor/independent variable; and age, sex, and sample source as covariates. Multiple hypothesis correction was performed with the Benjamini-Hochberg method using an FDR threshold of 10% (*q* < 0.1). Before visualization or correlation analysis, SOMAscan data were adjusted for age, sex, and sample source using the removeBatchEffect() function from the limma package (v3.44.3).

#### Analysis of MSD inflammatory marker data

Plasma concentration values (pg/ml) for each of the cytokines and related immune factors measured across multiple MSD assay plates were imported to R and combined, and analytes with >10% of values outside of detection or fit curve range were flagged. For each analyte, missing values were replaced with either the minimum (if below fit curve range) or maximum (if above fit curve range) calculated concentration per plate/batch and means of duplicate wells used for subsequent analysis. Extreme outliers were classified per karyotype and per analyte as measurements more than three times the interquartile range below or above the first and third quartiles, respectively, and excluded from further analysis. Differential abundance analysis for inflammatory markers measured by MSD was performed using mixed effects linear regression as implemented in the lmer() function from the lmerTest R package (v3.1-2), with log_2_-transformed concentration as the outcome/dependent variable, T21 status as the predictor/independent variable, age and sex as fixed covariates, and sample source as a random effect. Multiple hypothesis correction was performed with the Benjamini-Hochberg method using an FDR threshold of 10% (*q* < 0.1). Before visualization or correlation analysis, MSD data were adjusted for age, sex, and sample source using the removeBatchEffect() function from the limma package (v3.44.3).

##### Consensus clustering

For the consensus cluster described in Fig. 2, *z* scores were calculated from plasma concentration values (picograms per milliliter) for each of the inflammatory markers for samples from individuals with T21 and used as input to the ConsensusClusterPlus() function from the ConsensusClusterPlus package with 100-fold subsampling, Pearson as the distance measure, and agglomerative hierarchical clustering. Examination of the delta area plot indicated five clusters that gave a reasonable compromise between gains in cluster stability and number of clusters. The Mann-Whitney *U* test was used to test for differences in the distributions of DS IFN scores and other features for each cluster in comparison to cluster 1, using the wilcox_test() function from the rstatix package. Correction for multiple comparisons was performed using the Benjamini-Hochberg (FDR) approach.

#### Analysis of mass cytometry data

##### Preprocessing

Bead-based normalization via polystyrene beads embedded with lanthanides, both within and between batches, followed by bead removal was carried out as previously described using the Matlab-based Normalizer tool (*75*). Batched FCS files were demultiplexed using the Matlab-based Single Cell Debarcoder tool (*76*). Reference-based normalization of individual samples across batches against the common reference sample was then carried out using the R script BatchAdjust(). Last, for the analyses described here, CD3^+^CD19^+^ doublets were excluded, and Boolean gating for hematopoietic lineage (CD45-positive), non-granulocytic (CD66-low) cells was performed in CellEngine (CellCarta) and FCS files subsampled to 10,000 events per sample before export for subsequent analysis.

##### Unsupervised clustering

All 388 FCS files were imported into R as a flowSet object using the read.flowSet() function from the flowCore package (*77*). Next a SingleCellExperiment object was constructed from the flowSet object using the prepData() function from the CATALYST package (*78*), applying Arcsinh transformation with a cofactor of 5. Quality control and diagnostic plots were examined with the help of functions from the CATALYST and tidySingleCellExperiment packages.

Unsupervised clustering using the FlowSOM algorithm (*79*) was carried out using the cluster() function from the CATALYST package, with the grid size set to 10 × 10 to give 100 initial clusters, and a maxK value of up to 40 was explored for the subsequent meta-clustering. Clustering was rerun with multiple random seed values to ensure consistent results. Examination of delta area and minimal spanning tree plots (fig. S5A) indicated 30 meta-clusters that gave a reasonable compromise between gains in cluster stability and number of clusters.

##### *Visualization using* t*-distributed stochastic neighbor embedding*

Dimensionality reduction from 28 cell type markers to two dimensions was carried out using the runDR() function from the CATALYST package, with 500 cells per sample, and using several random seed values to ensure consistent results. Multiple values of the perplexity parameter were tested, with a setting of 440, using the formula Perplexity ~ *N*^(1/2)^ as suggested at https://towardsdatascience.com/how-to-tune-hyperparameters-of-tsne-7c0596a18868, providing a visualization with good agreement with the 30 clusters defined by FlowSOM.

##### Cell type classification

To aid in assignment of clusters to specific lineages and cell types, the MEM package (marker enrichment modeling) was used to call positive and negative markers for each cell cluster based on marker expression distributions across clusters. Manual review and comparison to marker expression histograms, as well as minimal spanning tree plots and *t*-distributed stochastic neighbor embedding (tSNE) plots colored by marker expression (e.g., fig. S5A), allowed for high-confidence assignment of most clusters to specific cell types (fig. S5B). Several clusters that were insufficiently distinguishable were merged into their nearest cluster based on the minimal spanning tree. Four clusters were excluded from further analysis (see fig. S5A), three because of low cell numbers (<0.01%) and one because of positivity for most markers. Relative frequencies for each cell type/cluster were calculated for each sample as a percentage of the total CD45^+^CD66^lo^ population.

##### Beta regression analysis

To identify cell clusters for which relative frequencies are associated with either T21 status or DS IFN scores in samples from individuals with T21, beta regression analysis was carried out using the betareg R package (v3.1-4), with each model using cell type cluster proportions (relative frequency) as the outcome/dependent variable and either T21 status or DS IFN score values as the independent/predictor variable, with adjustment for age and sex, and a logit link function. Extreme outliers were classified per karyotype and per cluster as measurements more than three times the interquartile range below or above the first and third quartiles, respectively (below Q1 − 3 × IQR or above Q3 + 3 × IQR), and excluded from beta regression analysis. Correction for multiple comparisons was performed using the Benjamini-Hochberg (FDR) approach. Effect sizes (as fold change in T21 versus control or per unit DS IFN score) for each cell type cluster were obtained by exponentiation of beta regression model coefficients. Fold changes from each model were visualized by overlaying on tSNE plots using ggplot2. For visualization of individual examples, data points were visualized as sina plots (separated by T21 status) or as *XY* scatterplots (for comparison to DS IFN scores) with points colored by local density using a custom function and overlaid with beta regression fit curves and 95% confidence intervals extracted from model objects using the ggemmeans() function from the ggeffects package (v1.1.0).

#### Analysis of plasma metabolomics and lipidomics data

Metabolite peak intensity data were imported to R. Metabolites with zero values were replaced with a random value sampled from between 0 and 0.5× the minimum nonzero intensity value for that metabolite. For downstream analysis, data were then normalized using a scaling factor derived by dividing the global median intensity value across all metabolites by each sample median intensity. Median normalization was chosen as it is simple to use, relies on few assumptions, and performs on par with more complex normalization techniques, such as linear regression, local regression, total intensity, average intensity, and quantile normalization, in reducing intragroup variation, and is one of the non–reference-based normalization methods used in the widely used MetaboAnalyst preprocessing module (*80*).

Extreme outliers were classified per karyotype and per analyte as measurements more than three times the interquartile range below or above the first and third quartiles, respectively, and excluded from further analysis. Differential abundance analysis for metabolites was performed using linear regression in R with log_2_-transformed relative abundance as the outcome/dependent variable; T21 status as the predictor/independent variable; and age, sex, and sample source as covariates. Multiple hypothesis correction was performed with the Benjamini-Hochberg method using an FDR threshold of 10% (*q* < 0.1). Before visualization or correlation analysis, metabolite data were adjusted for age, sex, and sample source using the removeBatchEffect() function from the limma package (v3.44.3).

#### Clustering and analysis of co-occurring conditions

Clinical metadata fields, including co-occurring conditions and past diagnoses, for individuals with T21 were filtered for conditions with at least 10 cases and controls and combined with matching DS IFN scores, resulting in 88 conditions eligible for clustering analysis. Pairwise sample-sample distances for this mixed data were then calculated using the Gower method as implemented in the daisy() function from the cluster package (v2.1.0). The resulting distance matrix was used as input for clustering using the PAM algorithm available in the pam() function, also from the cluster package. Silhouette width, a measure of measure of how similar an observation (here individual participant) is to its own cluster compared to other clusters, was then evaluated across a range of *k* values, with *k* = 2 clusters producing the highest average silhouette width. To visually compare occurrence of co-occurring conditions across the two clusters, the number of observed cases per cluster for each condition was divided by the expected number of cases (i.e., equal occurrence rates in each cluster) to give an observed/expected ratio that was plotted as a heatmap using the tidyHeatmap package.

Fisher’s exact test was used to test for unequal occurrence of each condition across clusters, using the tabyl() and fisher.test() functions from the janitor package (v2.0.1). The Mann-Whitney *U* test was used to test for differences in the distributions of DS IFN scores across clusters or between cases and controls, using the wilcox_test() function from the rstatix package. Correction for multiple comparisons was performed using the Benjamini-Hochberg (FDR) approach.

#### Longitudinal case study of tofacitinib treatment

A research participant in the HTP biobank received intermittent treatment with tofacitinib (Xeljanz; 5 mg of doses, once to twice a day) for alopecia areata with remarkable hair regrowth while on the medicine (*32*). Over the course of ~3 years, the participant provided research blood draws when on the medicine and during periods of voluntary treatment interruption. Blood draws were processed as described above for whole blood transcriptome and SOMAscan proteomics. RNA-seq and SOMAscan data were processed and analyzed as described above.

#### RNA-seq of murine tissues

Reads were demultiplexed and converted to FASTQ format using bcl2fastq v2.20.0.422. Data quality was assessed using FASTQC v0.11.5 and FastQ Screen v0.11.0. Filtering of low-quality reads was performed using bbduk from BBTools v37.99 and fastq-mcf from ea-utils v1.05. Alignment to the mouse GRCm38 reference genome index and Gencode M24 annotation GTF was carried out using HISAT2 v2.1.0. Alignments were sorted and filtered for mapping quality (MAPQ > 10) using SAMtools v1.5. Gene-level count data were quantified using HTSeq-count v0.6.1. RNA-seq data yield was a minimum of ~30 million raw reads. Differential gene expression was evaluated using DESeq2 v1.28.1 (*74*) with surrogate variables, determined using the svaseq() function from the sva package (version 3.46.0), as covariates to remove unwanted sources of variation, including sex and batch. Significance was set at *q* < 0.1 (10% FDR). GSEA (*73*) was carried out in R using the fgsea package (v 1.14.0), using Hallmark gene sets on a ranked list of log_2_-transformed fold changes.

## Notes

View/request a protocol for this paper from *Bio-protocol*.
