# Supplementary material for: Multidimensional definition of the interferonopathy of Down syndrome and its response to JAK inhibition
Source: Sci Adv. 2023 Jun 28;9(26):eadg6218. doi: 10.1126/sciadv.adg6218 (PMC10306300; doi:10.1126/sciadv.adg6218)
Supplement: Supplementary file 1 — Figs. S1 to S9 Legends for data S1 to S9 [file sciadv.adg6218_sm.pdf]

Science Advances
AAAS

Supplementary Materials for

Multidimensional definition of the interferonopathy of Down syndrome and its response to JAK inhibition

Matthew D. Galbraith *et al.*

Corresponding author: Joaquin M. Espinosa, joaquin.espinosa@cuanschutz.edu

Sci. Adv. **9**, eadg6218 (2023)
DOI: 10.1126/sciadv.adg6218

The PDF file includes:

Figs. S1 to S9
Legends for data S1 to S9

Other Supplementary Material for this manuscript includes the following:

Data S1 to S9

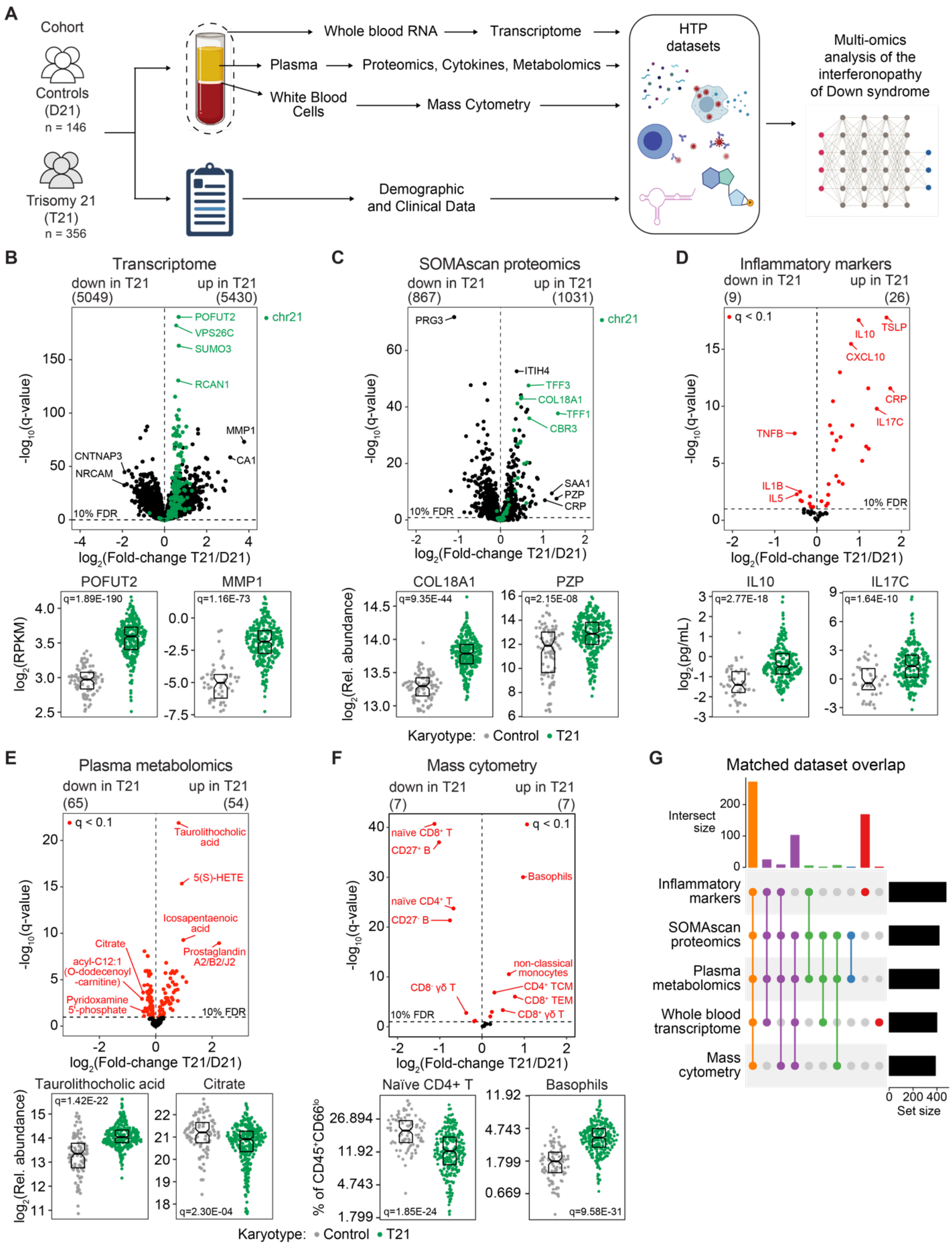
A
Cohort
Controls (D21) n = 146
Trisomy 21 (T21) n = 356
Whole blood RNA
Transcriptome
Plasma
Proteomics, Cytokines, Metabolomics
White Blood Cells
Mass Cytometry
Demographic and Clinical Data
HTP datasets
Multi-omics analysis of the interferonopathy of Down syndrome
B
Transcriptome
down in T21 (5049)
up in T21 (5430)
POFUT2
VPS26C
SUMO3
RCAN1
MMP1
CA1
CNTNAP3
NRCAM
chr21
10% FDR
-log10(q-value)
log2(Fold-change T21/D21)
POFUT2 q=1.89E-190
MMP1 q=1.16E-73
log2(RPKM)
C
SOMAscan proteomics
down in T21 (867)
up in T21 (1031)
PRG3
ITIH4
TFF3
COL18A1
TFF1
CBR3
SAA1
PZP
CRP
COL18A1 q=9.35E-44
PZP q=2.15E-08
log2(Rel. abundance)
Karyotype: Control T21
D
Inflammatory markers
down in T21 (9)
up in T21 (26)
q < 0.1
TSLP
IL10
CXCL10
CRP
IL17C
TNFB
IL1B
IL5
IL10 q=2.77E-18
IL17C q=1.64E-10
log2(pg/mL)
E
Plasma metabolomics
down in T21 (65)
up in T21 (54)
Taurolithocholic acid
5(S)-HETE
Icosapentaenoic acid
Prostaglandin A2/B2/J2
Citrate
acyl-C12:1 (O-dodecenoyl -carnitine)
Pyridoxamine 5'-phosphate
Taurolithocholic acid q=1.42E-22
Citrate q=2.30E-04
F
Mass cytometry
down in T21 (7)
up in T21 (7)
naïve CD8+ T
CD27+ B
naïve CD4+ T
CD27- B
CD8- γδ T
Basophils
non-classical monocytes
CD4+ TCM
CD8+ TEM
CD8+ γδ T
Naïve CD4+ T q=1.85E-24
Basophils q=9.58E-31
% of CD45+CD66lo
Karyotype: Control T21
G
Matched dataset overlap
Intersect size
Inflammatory markers
SOMAscan proteomics
Plasma metabolomics
Whole blood transcriptome
Mass cytometry
Set size

Fig. S1. Multiomics analysis of the interferonopathy of Down syndrome via the Human Trisome Project. (**A**) Overview of experimental approach. Research participants with trisomy 21 (T21) and euploid controls (D21) recruited into the Human Trisome Project (HTP) were characterized through matched multiomics analysis of blood samples and annotation of clinical data. (**B**) Volcano plot (upper) displaying the results of whole blood transcriptome analysis using DESeq2 from 304 participants with T21 versus 96 age- and sex-matched euploid controls (D21). RNAs encoded on chr21 in green. Sina plots (lower) comparing mRNA expression levels *POFUT2* and *MMP1*. (**C**) Volcano plot (upper) displaying the results of SOMAscan® proteomics on plasma samples from 316 participants with T21 versus 103 age- and sex-matched D21 controls. Proteins encoded on chr21 in green. Sina plots (lower) comparing relative abundance for example proteins COL18A1 and PZP. (**D**) Volcano plot (upper) displaying the results of inflammatory marker analysis on plasma samples from 346 participants with T21 versus 131 age- and sex-matched D21 controls. Sina plots (lower) comparing absolute concentrations for example proteins IL10 and IL17C. (**E**) Volcano plot (upper) displaying the results of plasma metabolomics analysis on plasma samples from 316 participants with T21 versus 103 age- and sex-matched D21 controls. Sina plots (lower) comparing relative abundance for example metabolites taurolithocholic acid and citrate. (**F**) Volcano plot (upper) displaying the results of mass cytometry analysis on blood samples from 292 participants with T21 versus 96 age- and sex-matched D21 controls. Sina plots (lower) showing the relative frequency (% among CD45+ $CD66^{lo}$ cells) for example immune clusters corresponding to naïve CD4+ T cells and basophils. **G.** Upset plot showing overlaps between matched blood samples across datasets. Horizontal dashed lines in volcano plots indicate q=0.1 (10% FDR). Boxes in sina plots represent interquartile ranges and medians, with notches approximating 95% confidence intervals.

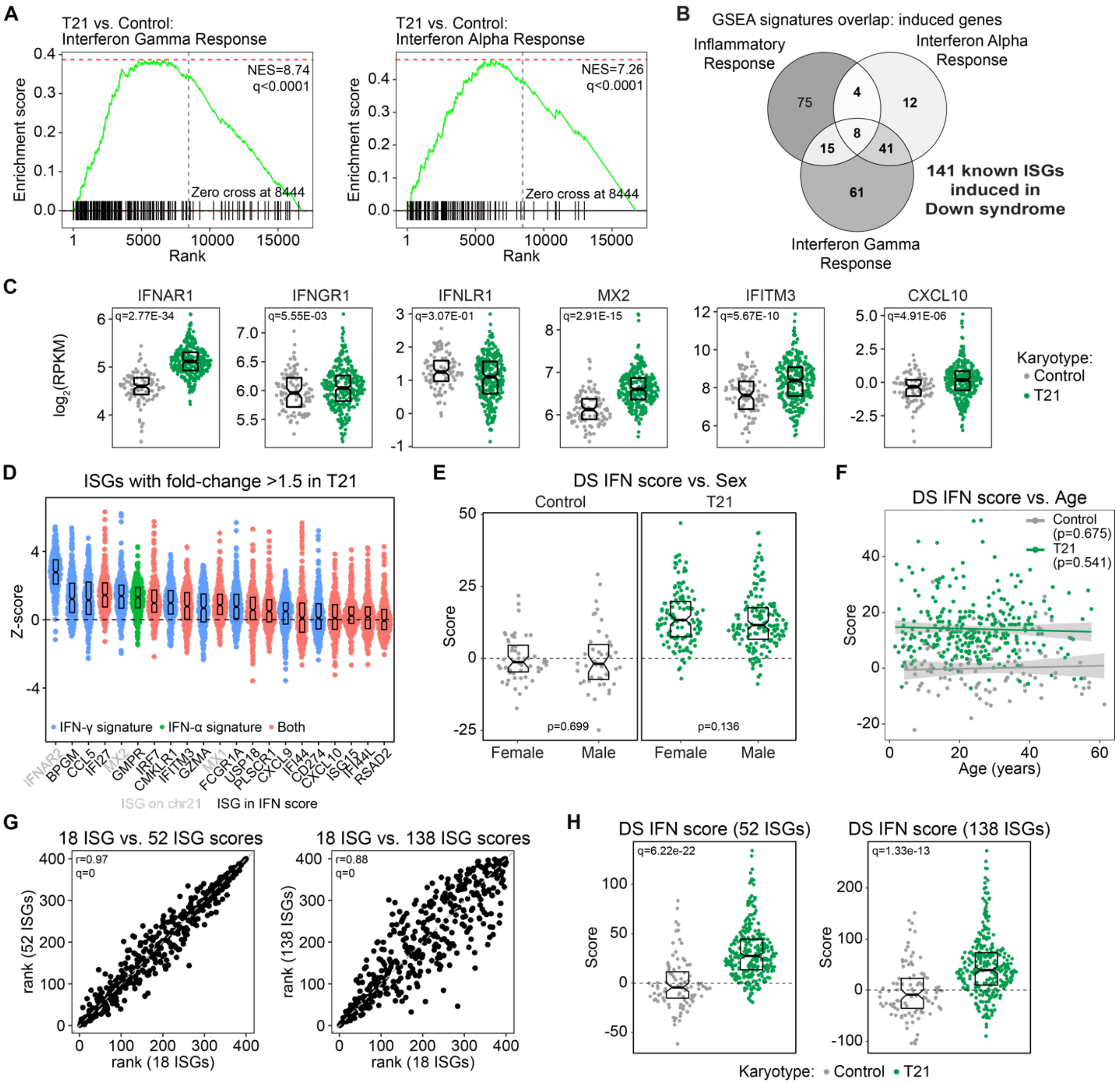

Fig. S2, related to Fig. 1. IFN transcriptional scores for the study of the interferonopathy of Down syndrome. (**A**) Gene set enrichment analysis (GSEA) plots for the Interferon Gamma and Alpha Response Hallmark gene sets. Green lines indicate cumulative enrichment score; black bars indicate gene set hits among all genes ranked by $\log_2$-fold-change (trisomy 21-T21/euploid controls). (**B**) Venn diagram displaying the overlap between genes induced in the whole blood transcriptome of individuals with T21 belonging to the Inflammatory Response, Interferon Gamma Response, and Interferon Alpha Response GSEA signatures, leading to the identification of 141 IFN-inducible genes (ISGs) elevated in Down syndrome. (**C**) Sina plots displaying mRNA expression of IFNRs and ISGs. (**D**) Sina plots displaying the relative expression of 21 ISGs significantly induced (q<0.1, 10% FDR) >1.5 fold in the whole blood transcriptome of individuals with T21. Values presented are Z-scores, calculated against the mean and standard deviation of euploid controls. Genes in grey (*IFNAR2*, *MX2*, *MX1*) are encoded on chr21 and were not used in calculating DS IFN scores. (**E**) Sina plots comparing distributions of DS IFN

scores in females and males for euploid controls (n=52/44) versus individuals with T21 (n=141/163). p-values from Mann-Whitney U test. (**F**) Scatter plot displaying DS IFN scores across the lifespan in euploid controls versus individuals with T21. p values from linear regression t-test. (**G-H**) Comparison of DS IFN scores calculated with the 18 ISGs selected in panel D versus 52 ISGs significantly induced (q<0.1, 10% FDR) at least 1.25-fold (G) and not encoded on chr21 or versus the complete set of 138 ISGs significantly induced (q<0.1, 10% FDR) in DS (H) that are not encoded on chr21. Pearson correlation scores and q-values are indicated in upper left. Boxes in sina plots represent interquartile ranges and medians, with notches approximating 95% confidence intervals.

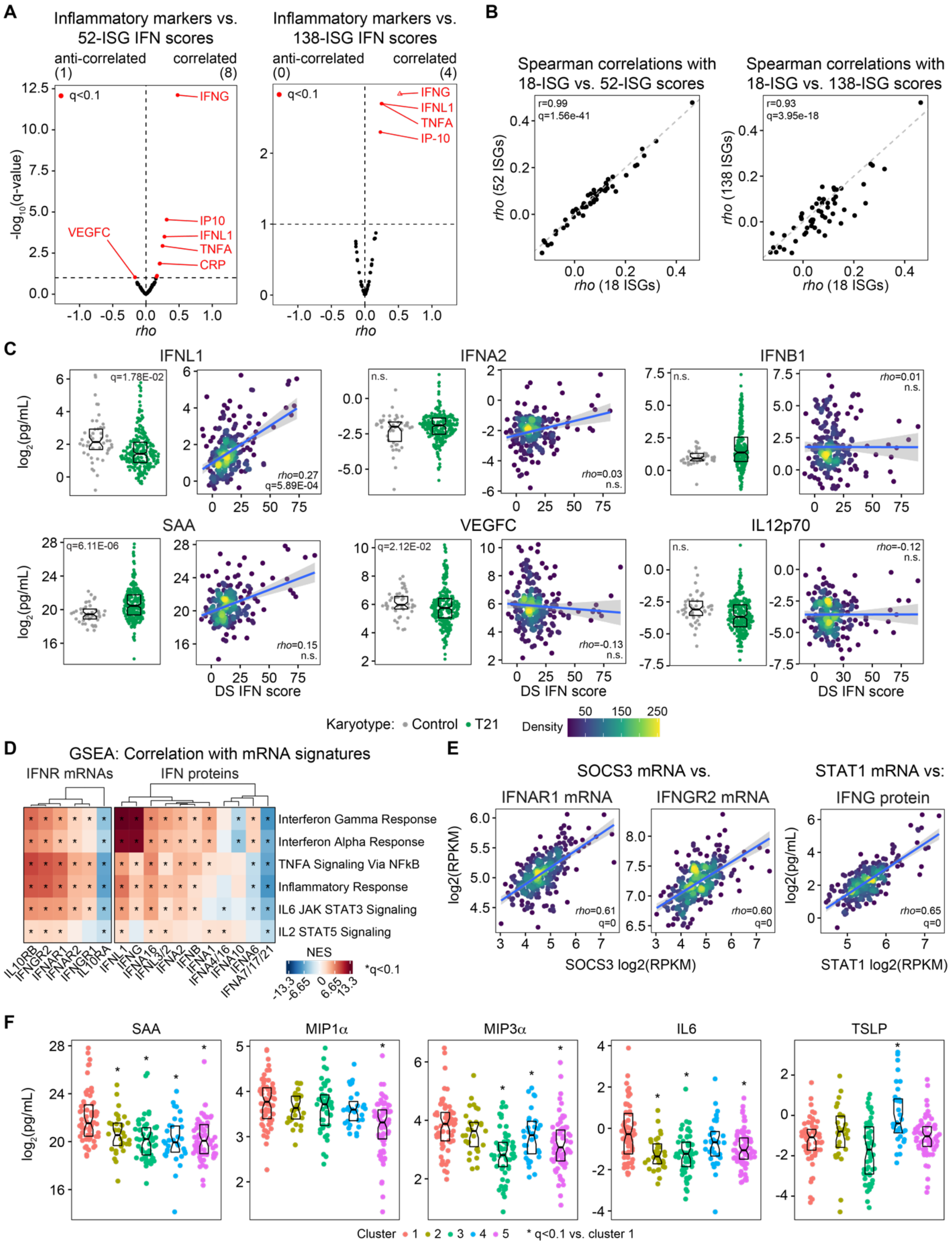

A
Inflammatory markers vs. 52-ISG IFN scores
anti-correlated (1)
correlated (8)
q<0.1
IFNG
IP10
IFNL1
TNFA
CRP
VEGFC
-log10(q-value)
rho
Inflammatory markers vs. 138-ISG IFN scores
anti-correlated (0)
correlated (4)
IFNG
IFNL1
TNFA
IP-10
B
Spearman correlations with 18-ISG vs. 52-ISG scores
r=0.99
q=1.56e-41
rho (52 ISGs)
rho (18 ISGs)
Spearman correlations with 18-ISG vs. 138-ISG scores
r=0.93
q=3.95e-18
rho (138 ISGs)
C
IFNL1
q=1.78E-02
rho=0.27
q=5.89E-04
IFNA2
n.s.
rho=0.03
IFNB1
rho=0.01
SAA
q=6.11E-06
rho=0.15
VEGFC
q=2.12E-02
rho=-0.13
IL12p70
rho=-0.12
log2(pg/mL)
DS IFN score
Karyotype: Control T21
Density
D
GSEA: Correlation with mRNA signatures
IFNR mRNAs
IFN proteins
Interferon Gamma Response
Interferon Alpha Response
TNFA Signaling Via NFkB
Inflammatory Response
IL6 JAK STAT3 Signaling
IL2 STAT5 Signaling
IL10RB
IFNGR2
IFNAR1
IFNAR2
IFNGR1
IL10RA
IFNL1
IFNG
IFNA16
IFNL3/2
IFNA2
IFNB
IFNA1
IFNA4/16
IFNA10
IFNA6
IFNA7/17/21
NES
*q<0.1
E
SOCS3 mRNA vs.
IFNAR1 mRNA
IFNGR2 mRNA
STAT1 mRNA vs:
IFNG protein
log2(RPKM)
rho=0.61
q=0
rho=0.60
q=0
rho=0.65
q=0
SOCS3 log2(RPKM)
STAT1 log2(RPKM)
log2(pg/mL)
F
SAA
MIP1α
MIP3α
IL6
TSLP
log2(pg/mL)
Cluster 1 2 3 4 5
* q<0.1 vs. cluster 1

Fig. S3, related to Fig. 2. IFN hyperactivity associates with a distinct pro-inflammatory immune subtype in Down syndrome. (**A**) Volcano plots for Spearman correlations of inflammatory markers versus the 52- and 138-ISG DS IFN scores. (**B**) Scatter plots comparing the pattern of correlations between inflammatory markers and DS IFN scores composed of 18 ISGs versus either 52 ISGs (left) or 138 ISGs (right). Pearson correlation scores and q-values are indicated in upper left. (**C**). Sina plots (left) displaying the effects of T21 on levels of various inflammatory markers and scatter plots (right) displaying their relationship with transcriptional DS IFN scores among those with T21 (with *rho* and q-values for Spearman correlation). Points are colored by density; blue lines represent linear model fit with 95% confidence intervals in grey. Significance defined as $q<0.1$ (10% FDR). (**D**) Heatmap displaying normalized enrichment scores (NES) from Gene Set Enrichment Analysis (GSEA) of Spearman correlations for the indicated IFNRs and IFNs against the whole blood transcriptome for inflammation-related Hallmark gene sets; asterisks indicate $q<0.1$ defined by GSEA with Benjamini-Hochberg correction. (**E**) Scatter plots displaying relationships between the indicated mRNAs and IFN mRNAs or IFNG protein among those with T21 (with *rho* and q-values for Spearman correlation). Points are colored by density; blue lines represent linear model fit with 95% confidence intervals in grey. Significance defined as $q<0.1$ (10% FDR). (**F**) Sina plots displaying distributions of transcriptional DS IFN scores and indicated inflammatory markers across the five immune subtypes identified in panel E. Asterisks indicate $q<0.1$ (10% FDR) for Mann-Whitney tests of each cluster against cluster 1). Boxes in sina plots represent interquartile ranges and medians, with notches approximating 95% confidence intervals.

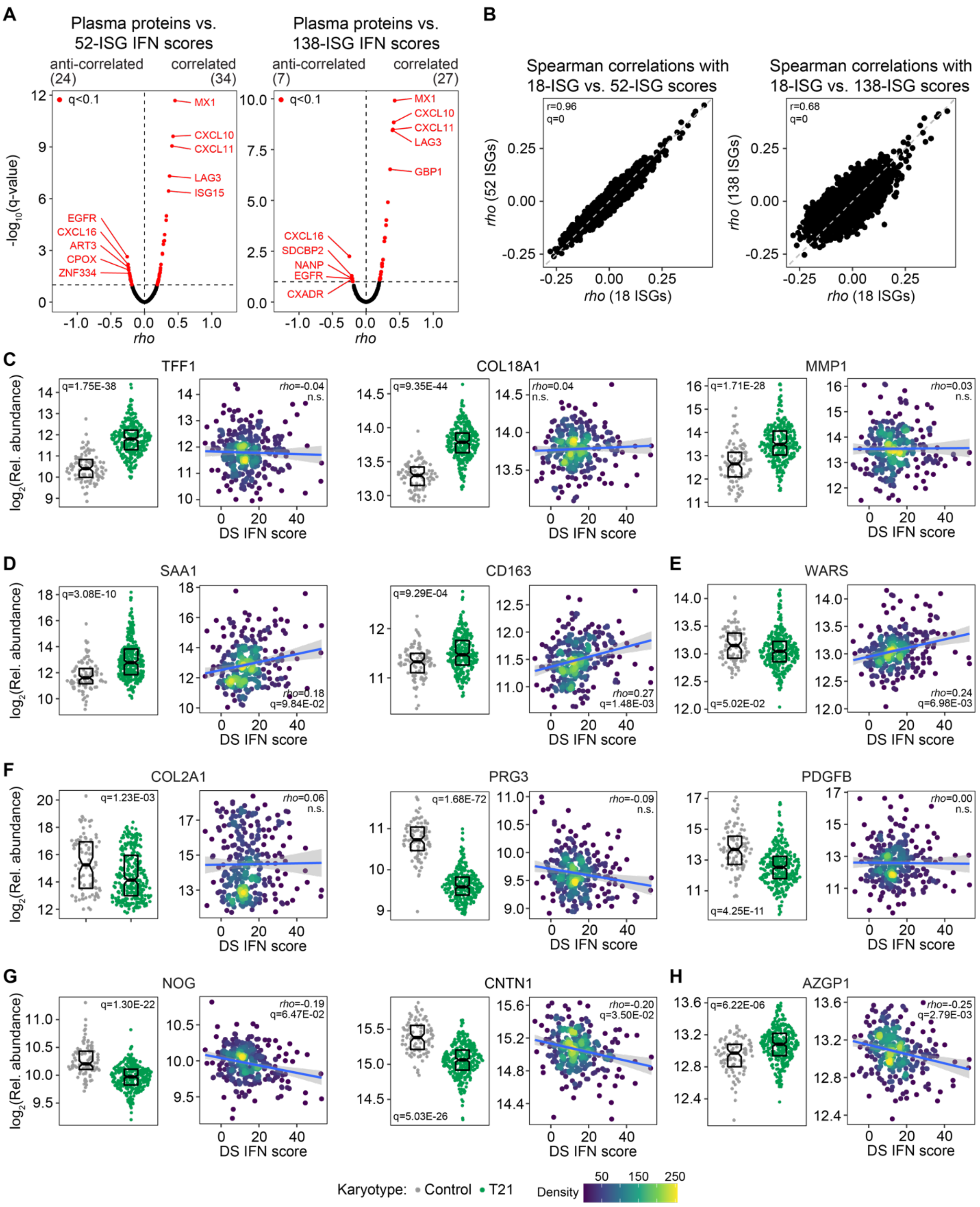

Fig. S4, related to Fig. 3. IFN hyperactivity reveals distinct classes of proteomics changes in Down syndrome. (**A**) Volcano plots for Spearman correlations of plasma proteins measured by the SOMAscan® platform versus the 52- and 138-ISG DS IFN scores. (**B**) Scatter plots comparing the pattern of correlations between plasma proteins and DS IFN scores composed of

18 ISGs versus either 52 ISGs (left) or 138 ISGs (right). Pearson correlation scores and q-values are indicated in upper left. (**C-H**) Sina plots (left) displaying the effects of T21 on levels of plasma proteins and scatter plots (right) displaying their relationship with transcriptional DS IFN scores among those with T21 (with *rho* and q-values for Spearman correlation). Points are colored by density; blue lines represent linear model fit with 95% confidence intervals in grey. Significance defined as $q<0.1$ (10% FDR). Boxes in sina plots represent interquartile ranges and medians, with notches approximating 95% confidence intervals.

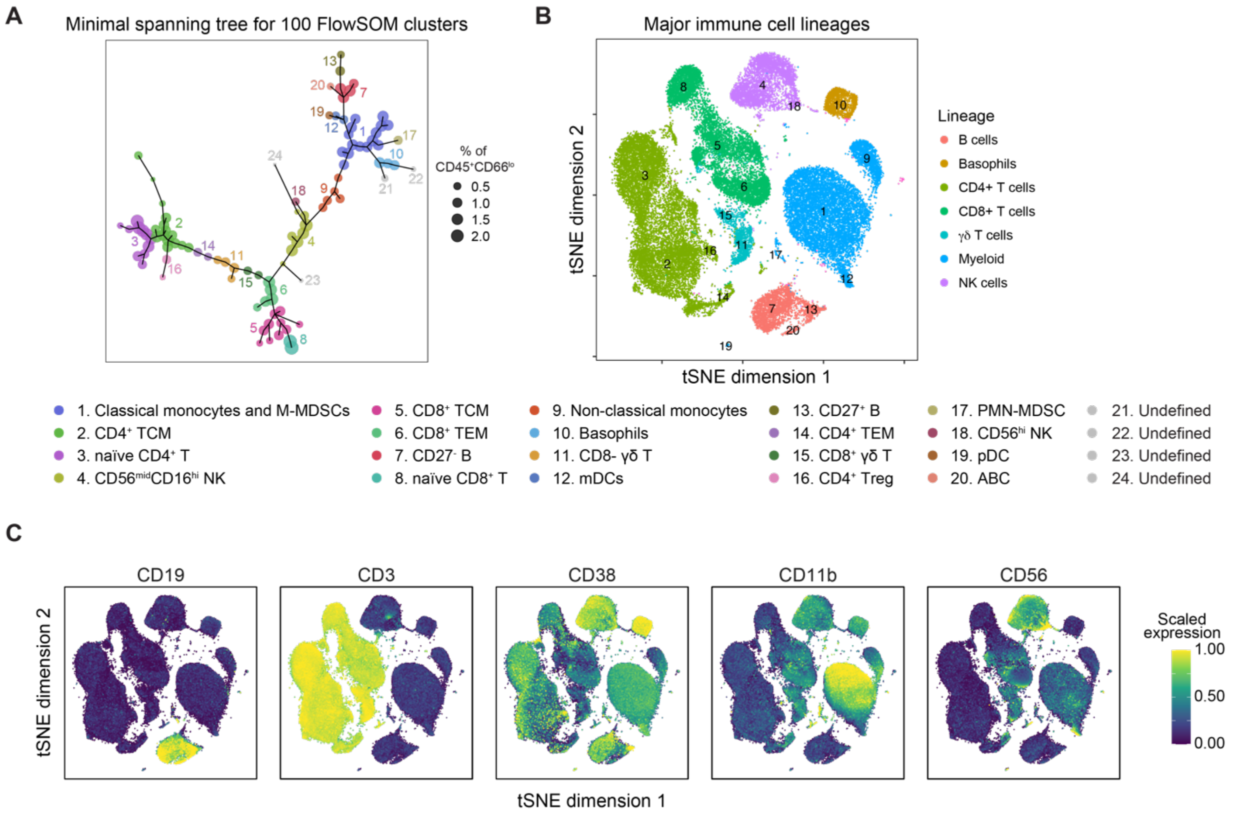

Fig. S5, related to Fig. 4. Immune mapping via mass cytometry in the Human Trisome Project. (**A**) Minimal spanning tree plot displaying the relationship of all 100 FlowSOM clusters identified among CD45+ CD66^{lo} cells after mass cytometry analysis of individuals with trisomy 21 (T21, n=292) versus euploid controls (D21, n=96). Metaclustering and manual review resulted in 20 main clusters for which cell types and major lineages were assigned based on marker expression. Four "undefined" clusters were excluded from further analysis. (**B**) t-Distributed Stochastic Neighbor Embedding (tSNE) plot displaying the 20 main immune clusters identified. (**C**) tSNE plots colored by scaled expression of example markers used to identify clusters corresponding to several of the major immune cell types. For all tSNE plots, only 500 cells per sample are displayed to reduce overplotting.

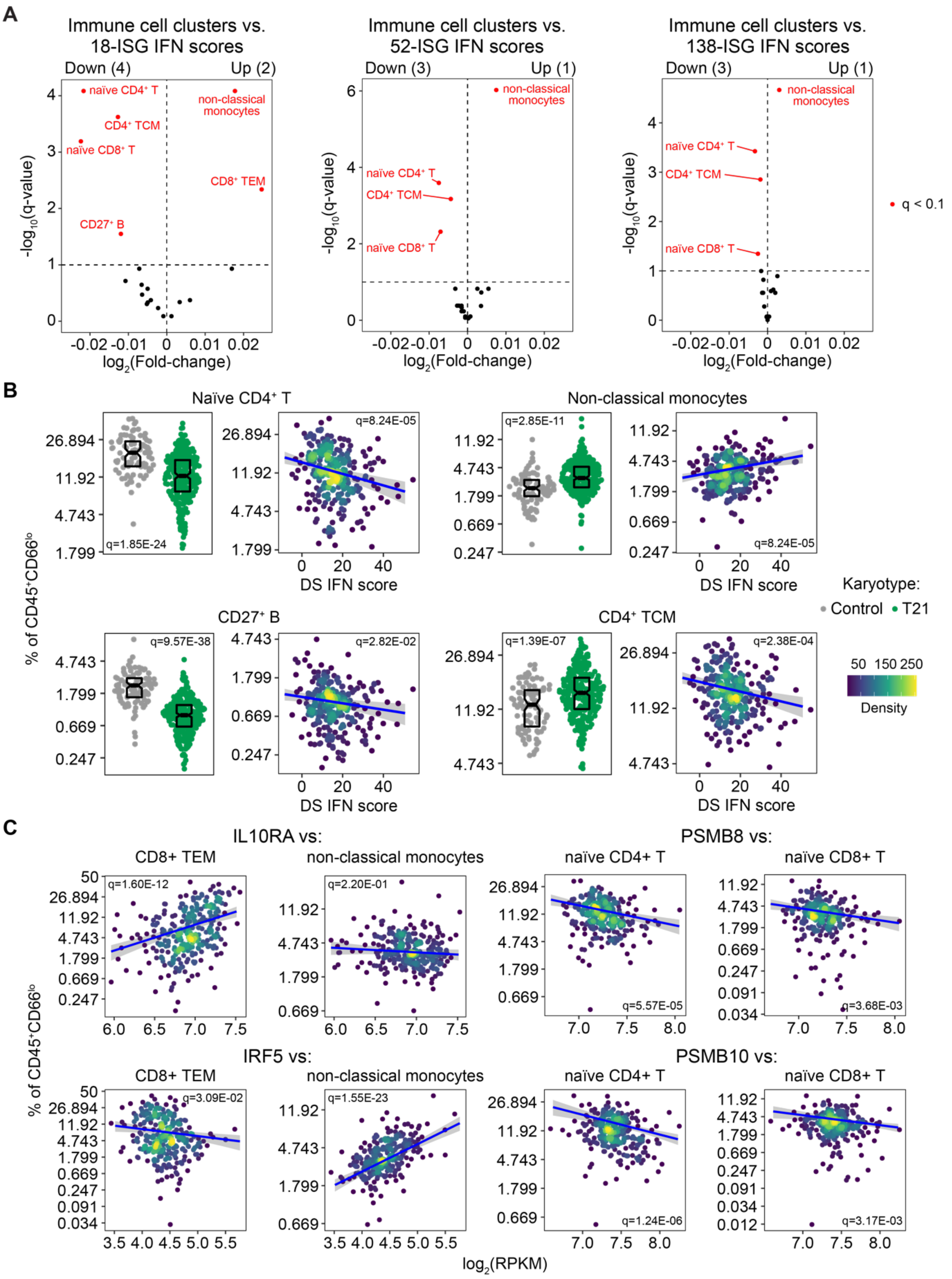
A
Immune cell clusters vs. 18-ISG IFN scores
Down (4)
Up (2)
naïve CD4+ T
CD4+ TCM
naïve CD8+ T
CD27+ B
non-classical monocytes
CD8+ TEM
Immune cell clusters vs. 52-ISG IFN scores
Down (3)
Up (1)
Immune cell clusters vs. 138-ISG IFN scores
-log10(q-value)
log2(Fold-change)
q < 0.1
B
Naïve CD4+ T
Non-classical monocytes
CD27+ B
CD4+ TCM
q=1.85E-24
q=8.24E-05
q=2.85E-11
q=8.24E-05
q=9.57E-38
q=2.82E-02
q=1.39E-07
q=2.38E-04
DS IFN score
% of CD45+CD66lo
Karyotype:
Control
T21
Density
C
IL10RA vs:
PSMB8 vs:
CD8+ TEM
non-classical monocytes
naïve CD4+ T
naïve CD8+ T
q=1.60E-12
q=2.20E-01
q=5.57E-05
q=3.68E-03
IRF5 vs:
PSMB10 vs:
q=3.09E-02
q=1.55E-23
q=1.24E-06
q=3.17E-03
log2(RPKM)

Fig. S6, related to Fig. 4. IFN hyperactivity associates with the extent of immune remodeling in Down syndrome. (**A**) Volcano plot displaying the Spearman correlations between transcriptional DS IFN scores (18, 52, or 138 ISGs) and frequencies of 20 major immune cell clusters identified in mass cytometry data from 277 individuals with trisomy 21. (**B**) Sina plots (left) displaying the effects of T21 on immune cell frequencies and scatter plots (right) displaying their relationship with transcriptional DS IFN scores among those with T21 (with q-values for beta regression). Points are colored by density; blue lines represent beta regression model fit with 95% confidence intervals in grey. Significance defined as q<0.1 (10% FDR). (**C**) Scatter plots displaying the relationship between relative frequencies of immune cell clusters and whole blood mRNA levels of the indicated ISGs among those with T21. Points are colored by density; blue lines represent beta regression model fit with 95% confidence intervals in grey. Significance defined as q<0.1 (10% FDR). Boxes in sina plots represent interquartile ranges and medians, with notches approximating 95% confidence intervals.

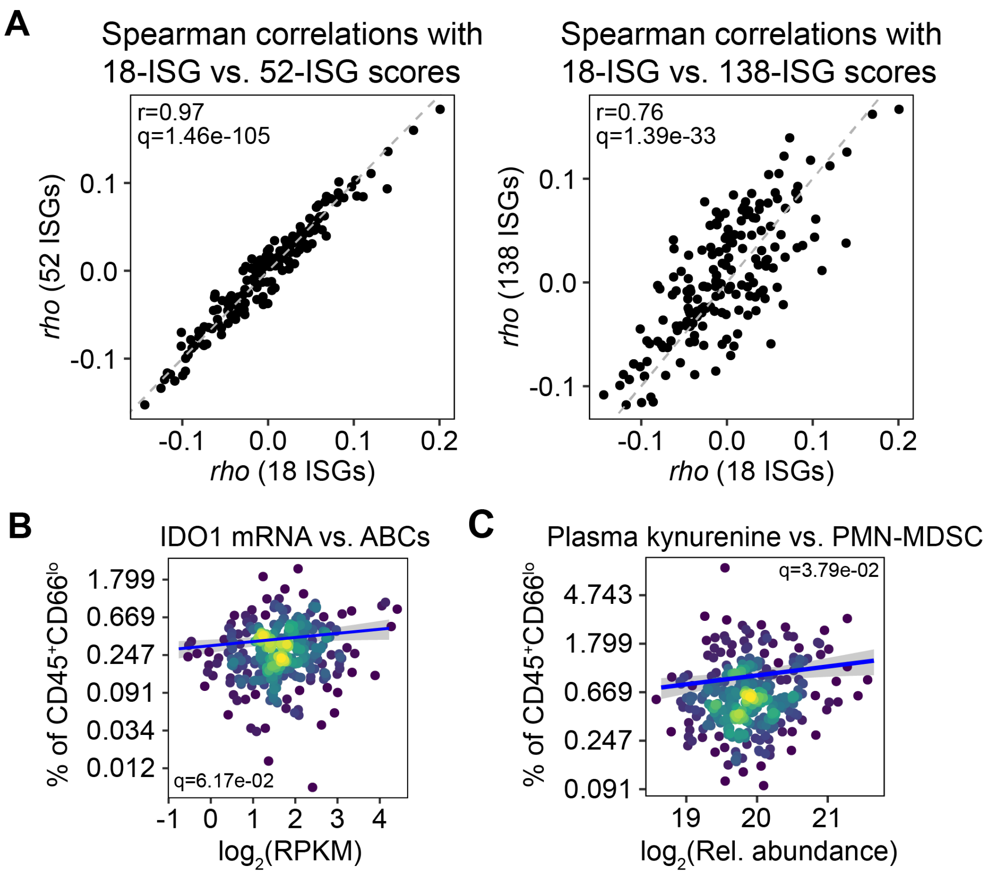

Fig. S7, related to Fig. 5. The metabolic signature of IFN hyperactivity in Down syndrome (**A**) Scatter plots comparing the pattern of correlations between plasma metabolites and DS IFN scores composed of 18 ISGs versus either 52 ISGs (left) or 138 ISGs (right). Pearson correlation scores and q-values are indicated in upper left. (**B**) Scatter plot displaying relationship between relative cluster frequency and IDO1 mRNA expression among those with T21 (with q-values for beta regression). Points are colored by density; blue lines represent beta regression model fit with 95% confidence intervals in grey. Significance defined as q<0.1 (10% FDR). (**C**) Scatter plot displaying relationship between relative cluster frequency and plasma kynurenine levels among those with T21 (with q-values for beta regression). Points are colored by density; blue lines represent beta regression model fit with 95% confidence intervals in grey. Significance defined as q<0.1 (10% FDR).

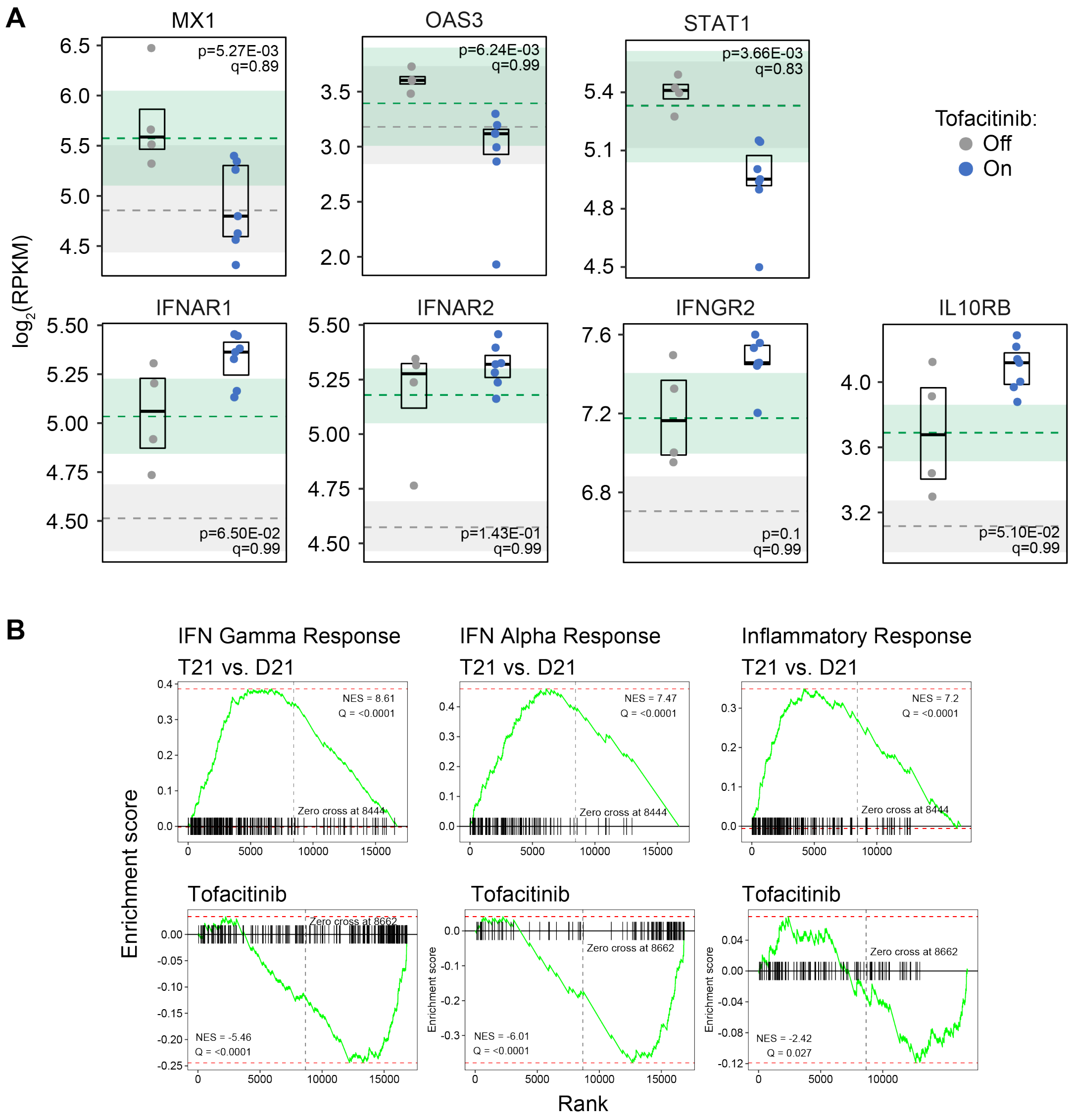

Fig. S8, related to Fig. 7. JAK inhibition normalizes IFN signatures and provides therapeutic benefit in Down syndrome. (**A**) Dot plots comparing mRNA expression levels for indicated genes in samples taken when the participant was off versus on tofacitinib treatment. Boxes represent interquartile ranges and medians for each group. Shaded areas represent the interquartile ranges for euploid controls (grey) and T21 samples (green), with dashed lines indicating median values. (**B**) Gene set enrichment analysis (GSEA) plots for the indicated Hallmark gene sets from MSigDB, comparing enrichment in T21/Control (upper) versus On/Off tofacitinib (lower). Green lines indicate cumulative enrichment score; black bars indicate gene set hits among all genes ranked by log_2-fold-change.

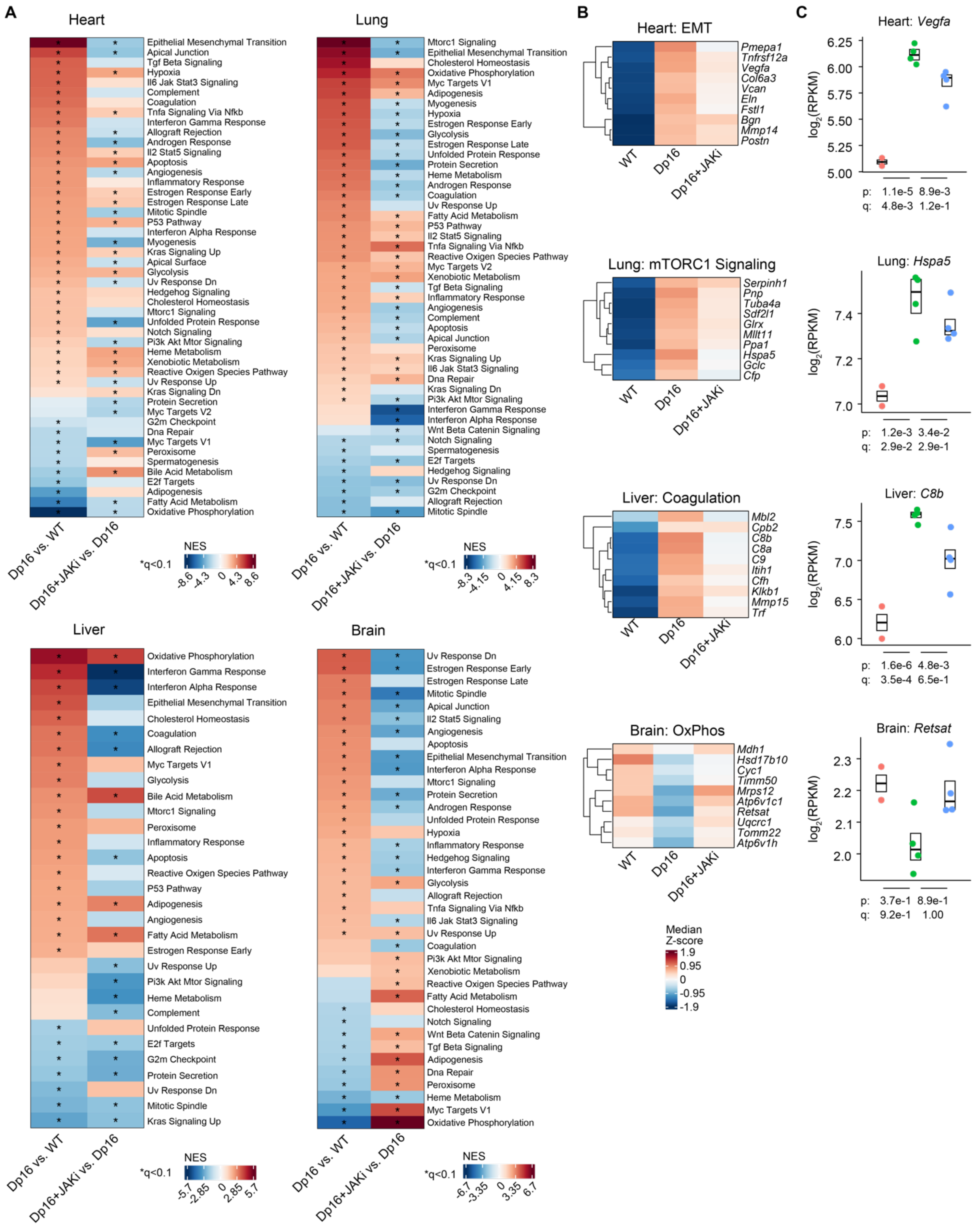

Fig. S9, related to Fig. 8. JAK inhibition attenuates global dysregulation of gene expression changes in the Dp16 mouse model of Down syndrome. (**A**) Heatmaps displaying normalized enrichment scores (NES) from Gene Set Enrichment Analysis (GSEA) of transcriptome fold-

changes for the indicated comparisons, sorted by NES for Dp16/WT comparison; asterisks indicate q<0.1 defined by GSEA with Benjamini-Hochberg correction. (**B**) Heatmaps representing median expression Z-scores per genotype (calculated from reads per kilobase per million (RPKM) values) for example genes from the indicated gene sets. (**C**) Sina plots for example genes from the corresponding gene sets in B; p- and q-values determined by DESeq2 with significance defined as q<0.1 after Benjamini-Hochberg correction.

Data S1. (separate file)

Cohort characteristics and multiomics analysis of the impacts of trisomy 21. (**A**) Cohort characteristics. (**B**) Whole blood RNA-seq DESeq2 results for T21 versus Control (euploid). (**C**) Plasma proteomics (SOMAscan) linear regression results for T21 versus Control. (**D**) Inflammatory markers (MSD) mixed effects linear regression results for T21 versus Control. (**E**) Plasma metabolomics (LCMS) linear regression results for T21 versus Control. (**F**) Mixed effects beta regression results for cell subpopulation proportions measured by mass cytometry (CyTOF) in T21 versus Control.

Data S2. (separate file)

Pathways analysis of transcriptome data and interferon scores. (**A**) Gene Set Enrichment Analysis (GSEA) of whole-blood transcriptome data by Karyotype; using log2(Adjusted FoldChange) from DESeq2 analysis as the ranking metric and "Hallmark" gene sets from MSigDB. (**B**) RNA-based DS IFN scores calculated as the sum of 18 gene Z-scores calculated against the mean and standard deviation of euploid controls for 400 HTP participants.

Data S3. (separate file)

Associations between inflammatory markers and IFN scores. (**A**) Results for Spearman correlations between inflammatory markers measured by multiplex immunoassay (MSD) and DS IFN scores for individuals with T21.

Data S4. (separate file)

Proteomics analysis of interferon hyperactivity in Down syndrome. (**A**) Gene Set Enrichment Analysis (GSEA) of plasma proteome (SOMAscan) data by Karyotype; using log2(FoldChange)*-log10(p-value) from linear regression analysis as the ranking metric and "Hallmark" gene sets from MSigDB. (**B**) Results for Spearman correlations between aptamers measured by SOMAscan and DS IFN scores for individuals with T21. **C.** GSEA using Spearman correlation coefficients (*rho*) vs DS IFN score as the ranking metric and "Hallmark" gene sets from MSigDB.

Data S5. (separate file)

Mass cytometry analysis of the interferon hyperactivity in Down syndrome. (**A**) Mixed effects beta regression results for cell subpopulation proportions measured by mass cytometry (CyTOF) versus DS IFN scores for individuals with T21. (**B**) Mixed effects beta regression results for cell subpopulation proportions measured by mass cytometry (CyTOF) versus mRNA expression levels of interferon-stimulated genes (ISGs) in individuals with T21.

Data S6. (separate file)

Metabolomics analysis of the interferon hyperactivity in Down syndrome. (**A**) Results for Spearman correlations between metabolites measured by LCMS and DS IFN scores for individuals with T21. (**B**) Mixed effects beta regression results for cell subpopulation proportions

measured by mass cytometry (CyTOF) versus *IDO1* mRNA expression levels in individuals with T21. (**C**) Mixed effects beta regression results for cell subpopulation proportions measured by mass cytometry (CyTOF) versus plasma kynurenine levels in individuals with T21.

Data S7. (separate file)

Transcriptome and proteomics analysis of JAK inhibition in Down syndrome. (**A**) Whole blood RNA-seq DESeq2 results for "On" versus "Off" tofacitinib. (**B**) Plasma proteomics (SOMAscan) linear regression results for "On" versus "Off" tofacitinib.

Data S8. (separate file)

Transcriptome analysis of JAK inhibition in a mouse model of Down syndrome. (**A**) Murine heart tissue RNA-seq: DESeq2 Results for Dp16 versus WT (C57/BL6). (**B**) Murine lung tissue RNA-seq: DESeq2 Results for Dp16 versus WT (C57/BL6). (**C**) Murine liver tissue RNA-seq: DESeq2 Results for Dp16 versus WT (C57/BL6). (**D**) Murine brain tissue RNA-seq: DESeq2 Results for Dp16 versus WT (C57/BL6). (**E**) Murine heart tissue RNA-seq: DESeq2 Results for Dp16+JAKi versus WT Dp16+Vehicle. (**F**) Murine lung tissue RNA-seq: DESeq2 Results for Dp16+JAKi versus WT Dp16+Vehicle. (**G**) Murine liver tissue RNA-seq: DESeq2 Results for Dp16+JAKi versus WT Dp16+Vehicle. (**H**) Murine brain tissue RNA-seq: DESeq2 Results for Dp16+JAKi versus WT Dp16+Vehicle. (**I**) Murine heart tissue RNA-seq: DESeq2 Results for Dp16+JAKi versus WT+Vehicle. (**J**) Murine lung tissue RNA-seq: DESeq2 Results for Dp16+JAKi versus WT+Vehicle. (**K**) Murine liver tissue RNA-seq: DESeq2 Results for Dp16+JAKi versus WT+Vehicle. (**L**) Murine brain tissue RNA-seq: DESeq2 Results for Dp16+JAKi versus WT+Vehicle. (**M**) Gene Set Enrichment Analysis (GSEA) for mouse heart. (**N**) Gene Set Enrichment Analysis (GSEA) for mouse lung. (**O**) Gene Set Enrichment Analysis (GSEA) for mouse liver. (**P**) Gene Set Enrichment Analysis (GSEA) for mouse brain.

Data S9. (separate file)

List of antibodies and key reagents used in this study.
